# Exploring similarities and differences in how researchers and young people understand key terms in youth mental-health research

**DOI:** 10.1057/s41599-025-05809-5

**Published:** 2025-09-29

**Authors:** Raginie Duara, Georgia Pavlopoulou, Siobhan Hugh-Jones, Nicola Shaughnessy, Ruth Herbert, Sylvan Baker, Emma Williams, Edmund Sonuga-Barke, Kamaldeep Bhui, Anna Mankee-Williams, Paul Cooke

**Affiliations:** 1 University of Leeds, Leeds, United Kingdom; 2 University College London, London, United Kingdom; 3 University of Kent, Canterbury, United Kingdom; 4 City St George’s, University of London, London, United Kingdom; 5 Royal Central School of Speech and Drama, London, United Kingdom; 6 University of Surrey, Guildford, United Kingdom; 7 King’s College London, London, United Kingdom; 8 University of Oxford, Oxford, United Kingdom; 9 Falmouth University, Falmouth, United Kingdom

**Keywords:** Health humanities, Psychology

## Abstract

A lack of a shared understanding of key terms is acknowledged as a significant barrier to interdisciplinary research. This paper examines the ways in which a broadly interdisciplinary team of academics and youth co-researchers involved in mental health research interpreted a number of research and mental health terms that are central to their work in order to understand conceptual differences in how different stakeholder groups approach these terms. Data was collected in four phases (interviews, written responses, and two participatory ‘living labs’) and was analysed using reflexive thematic analysis. Results revealed a wide disparity in the way participants understood key terms (including: ‘research’, ‘data’, ‘loneliness’, ‘safe space’ and ‘resilience’). Our study highlights the need for more inclusive approaches to mental health research, where diverse perspectives and lived experiences inform both methodology and practice from the outset. In conclusion we suggest a new framework (the EQUITY framework) as a tool to operationalise these findings.

## Introduction

Youth Mental health research is an increasingly interdisciplinary endeavour, the research community understanding that the issues young people face cannot be addressed adequately from a single disciplinary perspective (Handron et al., 2001; Bailey, 2012; Colizzi et al., 2020; O’Hara et al., 2019). Nor can they be addressed, as the literature also widely accepts, without acknowledging the views of young people themselves as ‘experts in their own lives’ (Coyne and Carter, 2024, p.5). Engaging in interdisciplinary research with young people can, however, be difficult. On the one hand, multi-perspectival, interdisciplinary studies can be impeded by ‘disagreement among scholars with respect to some of the vocabulary used for fundamental concepts’ (Newman, 2024), this lack of agreement leading to ‘cognitive barriers which contribut[e] to failures in interdisciplinary research’ (MacLeod, 2018). On the other, as Coyne and Carter go on to argue, valuing the expertise of young people, and treating them as equitable partners, even co-researchers, within a study ‘challeng[es] traditional research dynamics and adult-youth power dynamics’ (2018, p.5; see also Leman et al., 2024). If this challenge is not adequately addressed, involving young people in the research process might ultimately cause more problems than it solves. It can, for example, lead to young people feeling exploited by researchers for their knowledge, or even to the potential exacerbation of the issue the research project is ostensibly seeking to address (Mawn et al., 2016; Totzeck et al., 2024; Schmid and Garrels, 2024; Wyatt et al., 2024).

To support interdisciplinary mental health research with young people, our paper explores the competing ways in which a group of academics from across the disciplinary landscape (from the clinical and social sciences to the arts and humanities) and young people actively involved in a mental health project as co-researchers understand some of the key terms that define their collective work. The results reported here form a sub-strand of Project CREATE (Creating Research Ecologies to Advance Transdisciplinary lEarning: Arts-based programs and the study of adolescent loneliness) which aims to progress methodologies for youth-collaborative, interdisciplinary mental health research. Funded by the UK Arts and Humanities, Economic and Social Research and Medical Research Councils, CREATE focussed on researching arts-based practices (ABP) for youth mental health, taking adolescent loneliness its case study. ABP can benefit young people living with mental health conditions (e.g. anxiety, depression) and associated challenges, including loneliness (APPG Arts, Health and Wellbeing 2017; Fancourt et al., 2019; Lim et al., 2020; Tymoszuk et al., 2020). However, interdisciplinary research on ABPs for youth mental health regularly encounters certain methodological challenges, not least the issue described above (and upon which we focus in this paper) namely the lack of a shared understanding of key terms that ‘make sense’ for all the stakeholders involved (Daykin et al., (2017); Kwan and Rickwood, 2015). The study we report here does not focus on the role of ABP per se for youth mental health. However, we did utilise ABP (and specifically an arts-based Living Lab approach, discussed further below) as part of our data collection methodology.

## Methods

Ethical approval was obtained from the Arts, Humanities and Cultures Ethics Committee at University of Leeds. No ethical concerns were raised during the research.

**Fig. 1:**
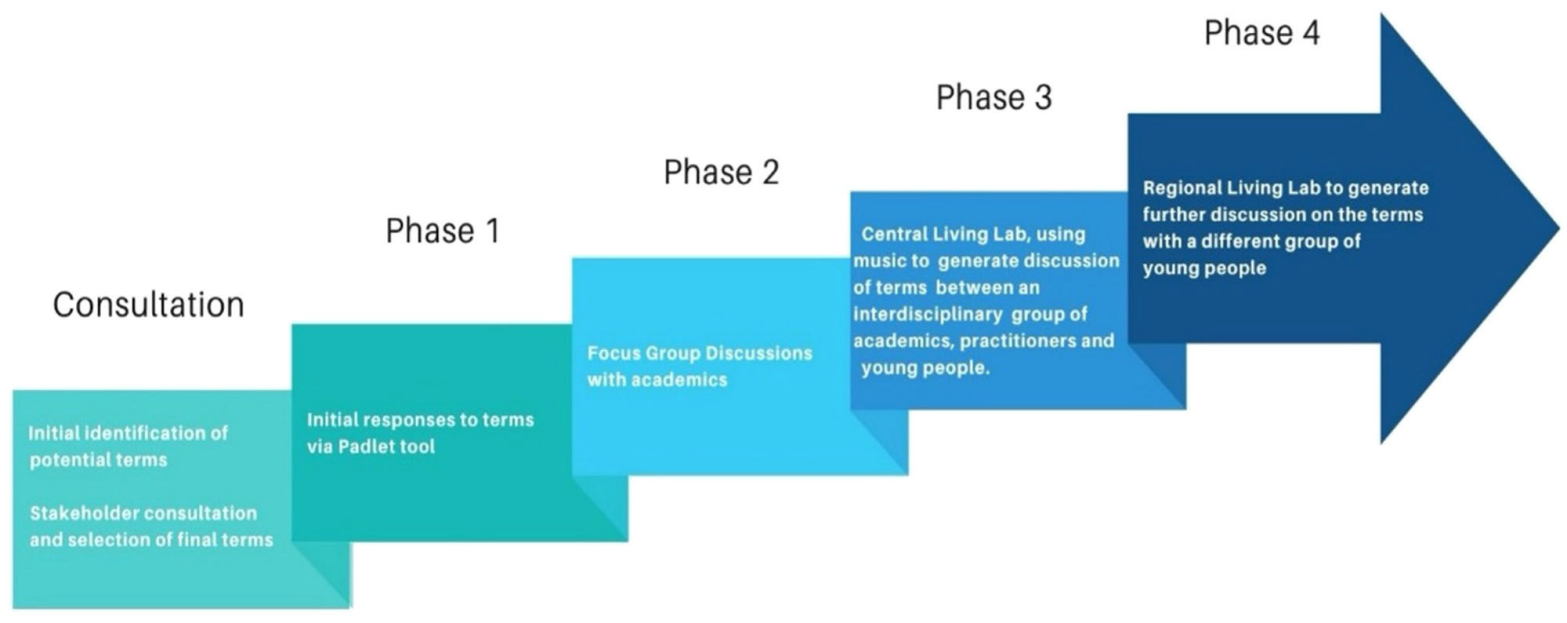
Iterative Stepped approach to data collection used in our project.

We employed an iterative, stepped approach to data collection, as illustrated in Fig. 1. This was a Delphi-informed approach, which is widely used to elicit expert consensus in complex or emerging areas (Hsu and Sandford, 2007). Although our method diverged from the classical Delphi model—most notably in not using multiple anonymous feedback rounds—it retained key features such as structured stakeholder engagement, iterative input, and prioritization based on expert judgment.

### Consultation

Step 1: The project’s principal investigator (PC) and postdoctoral researcher (RD) generated a small list of terms stemming from their experience of arts-based, interdisciplinary youth mental health research projects, differentiating between terms connected specifically to mental health and generic research terms. These were sent to five interdisciplinary research team members who were asked to prioritise the terms and add others they had encountered and found problematic in their own work.

Step 2: This involved approaching members from a cognate project (ATTUNE, funded by the same commissioning call) and project CREATE’s associated arts-and-health partners (COMIC and Artswell) to take part in a survey. Stakeholders were also asked to share the study call with their networks. The survey asked respondents to choose 5 research-related and 10 mental health-related terms from the list generated during Step 1 which they perceived as particularly challenging in interdisciplinary, youth mental health research. A total of 28 mental health experts, including academic researchers, mental health and arts-based practitioners, responded (see supplementary materials for participants’ roles/identity). Survey outcomes were discussed with the project team and the 14 most frequently chosen terms were included for further exploration, as shown in Table 1.

**Table 1:** Outcome of Step 2—terms (*n* = 14) for further exploration.

Mental health related terms	Research related terms
Resilience	Research
Mental health	Data
Stigma	Evidence
Wellbeing	
Empowerment	
Loneliness	
Connection	
Coping	
Community	
Safe space	
Agency	

### Phases 1–4

#### Participants

Young people (functioning in this project as co-researchers) and academics participated in Phases 1–4. Although it should be noted from the outset that these labels (academics, young people) belie considerable heterogeneity. We recruited young people from CREATE’s youth advisory group (YPAG) and regional ‘Living Lab’. Table 2 shows some participant demographics. 4 of the young participants (2 from our YPAG and 2 from our regional Living Lab—see below for further explanation) identified as neurodivergent, contributing to the inclusivity and diversity of our sample and the perspectives generated.

**Table 2:** Demographics of the young people involved in Phases 1–4.

YPAG members				Regional Living Lab participants			
Participant	Age	Gender	Ethnicity	Participant	Age	Gender	Ethnicity
Y1	18	Female	White British	R1	11	Male	White British
Y2	21	Female	White British	R2	14	Male	White British
Y3	20	Female	Asian British Pakistani	R3	14	Female	White British
Y4	19	Female	Asian British Bangladeshi	R4	11	Female	White British
Y5	19	Female	Asian British Indian	R5	15	Male	White British
Y6	19	Female	White British	R6	11	Male	White British
Y7	19	Male	Mixed White and Asian	R7	11	Male	White British
Y8	20	Female	White: Other background	R8	14	Female	White British
Y9	18	Male	Asian British Bangladeshi	R9	14	Male	White British
Y10	16	Female	Asian British Indian	R10	13	Male	White British
Y11	16	Female	Asian British Indian				
Y12	14	Male	Asian British Indian				
Y13	16	Female	Asian British Indian				
Y14	12	Female	Asian British Indian				
Y15	12	Female	Mixed White & Black Caribbean				
Y16	16	Female	Asian British Indian				
Y17	15	Female	Mixed White and Asian				
Y18	12	Male	Black & Black Caribbean				
Y19	14	Female	Asian British Indian				
Y20	14	Female	Asian British Indian				

We recruited 10 academics for Phase 2. Table 3 shows their key details. Two of this group identified as neurodivergent. To uncover some of the nuances between academics from very different disciplines while maintaining confidentiality, we have collected them into broad disciplinary groupings:

**Table 3:** Key details of academics (*n* = 10) participating in Phase 3.

Participant	Gender	Ethnicity	Academic position	Department/School	Broad disciplinary grouping
A1	Female	White British	Professor	Theatre and Performance	Arts and Humanities (AH)
A2	Female	White British	Senior lecturer	Music and Audio Technology, School of Arts	Arts and Humanities (AH)
A3	Male	White British	Professor	Film Studies	Arts and Humanities (AH)
A4	Female	White: Other background	Associate Professor	Brain Sciences	Clinical (C)
A5	Female	White British	Research Assistant	Health Sciences	Social Science (S)
A6	Female	White British	Postdoctoral Researcher	Psychiatry	Clinical (C)
A7	Female	White British	Associate Professor	Health Psychology	Social Science (S)
A8	Male	White British	Professor	Developmental Psychology, Psychiatry and Neuroscience	Clinical (C)
A9	Male	Black British	Senior Lecturer	Community Performance/Applied Theatre	Arts and Humanities (AH)
A10	Female	White British	Lecturer	Developmental Psychology	Social Science (S)

#### Data Collection

We gathered opinions and perspectives on the meanings of each of the selected terms (Table 1) through 4 phases of data collection, each using a different method: 1. responses via the Padlet tool, 2. focus groups, 3. a central Living Lab, and 4. a regional Living Lab. Table 4 outlines the data generated from each phase. Outcomes of each phase informed the next.

**Table 4:** Showing data sources across the four phases.

Participants	Phase 1	Phase 2	Phase 3	Phase 4
**YPAG**	Padlets with terms and responses (separate for 10–17- and 18–24-year-olds)		1: Each chose a song to represent their selected term and explained their connection in individual recordings. 2: Grouped terms and developed musicals; group discussions followed	
**Academics**	Padlet with terms and responses	Focus group discussion		
**YP in regional Living Lab**				1: Grouped terms and added explanations. 2: Created a song with lyrics entitled ‘Safe space’

#### Procedure

##### Phase 1 responses via Padlet

we sought perspectives on each term via the Padlet tool. Participants were asked to write answers to the question ‘What do these terms, related to research and mental health, mean to you?’ There was a separate Padlet for academics and for youth co-researchers and all responses were anonymous. Two separate sessions were conducted with young people from the YPAG, one from the 10–17 age group and one with the 18–24 group, to support their completion of the Padlet. After these sessions, links to the Padlet were sent to the youth co-researchers, in case they wanted to add more responses. They were given an additional month for this process. Academics were simply sent a link to the Padlet and asked to respond directly on the tool. The written responses formed part of our final analysis (see results section) and informed Phases 2 and 3.

##### Phase 2 focus group

An information sheet and consent form were emailed to the 28 participants who helped identify the terms, inviting them to participate in a focus group. We conducted a recorded, two-hour online focus group discussion, driven by the Padlet responses, which were always available on screen. Phase 1 academics were invited to expand or explain their Padlet responses. Analytic outcomes of this Phase are presented separately in the results section.

##### Phase 3 central Living Lab

The aim of Phase 3 was to gather further perspectives from the youth co-researchers and to put these into dialogue with other members of the research team via a ‘Living Lab’. A Living Lab refers to practices that are user driven and involve all parties in co-creation. They are designed to engage with lived and/or place-based experiences, generating a multidisciplinary ecosystem whereby a complex problem can be studied from different disciplinary perspectives using the group’s collective intelligence (Compagnucci et al., 2021). In the sciences, it is conceptualised as ‘a research methodology for sensing, prototyping, validating and refining complex solutions in multiple and evolving real-life contexts’, commonly used, for example, in ‘Ambient Intelligence’ research (Robben et al., 2012). In the arts, this same ethos is brought to bear on a given issue, using a variety of ABPs as the vehicle for discussion (Findlay and Filipowicz, 1986). In our case we used music in a variety of ways (both listening to pre-recorded and creating new music) to bring young people’s perspectives on our terms into dialogue with other stakeholders in this study.

Supplementary Materials detail the structure of our Living Lab. In brief, the Living Lab took place in a music studio in Leeds. Academics (n = 4) from the project team (from theatre and performance, media studies and music psychology) took part in the Living Lab discussions. Music-making in the Living Lab was facilitated by two music-related arts-based practitioners. Young people were asked to engage in two tasks. Task 1 invited participants to select a Table 1 term and a song they knew that could represent this term for them. Songs were chosen from YouTube, Spotify and Apple Music. Task 2 invited young people to work in groups to create ‘families’ of terms which they subsequently used to craft their own musical compositions reflective, for them, of the essence of these ‘families’. These compositions were then performed to the rest of the Living Lab. The performances were followed by group discussions, which were audio-recorded and subsequently transcribed, capturing participants’ views on the musical compositions and how these related to their chosen group of terms, as well as how the academics and facilitators responded to the young people’s musical rendering of these terms.

##### Phase 4 regional Living Lab

The aim of the one-day, music-based regional Living Lab was to probe Phase 3 findings in collaboration with youth researchers from a different part of the UK (Falmouth. See supplementary materials for workshop details) and led by experienced arts-practitioners. In a similar fashion to the central Living Lab, young people were invited to take part in 2 tasks. Task 1 invited the young people involved to group the 14 terms (Table 1) into ‘families’ based on their understanding of what the terms meant. Task 2 invited them to choose what they considered to be the most important ‘family’ and collectively to produce a song to represent the terms involved. The song was performed and was followed by a discussion, which was audio-recorded and subsequently transcribed.

## Analysis

### Data preparation

Phase 1 data (academic contribution to Padlet) and Phase 2 (focus group) were combined as representing meanings academics assigned to the selected terms. Phase 1 data (youth contribution to Padlet) and Phases 3 and 4 (Living Labs) were combined as representing meanings young people attributed to the terms.

### Analysis procedure

We followed Braun and Clarke’s (2021) steps for reflexive thematic analysis, with a focus on capturing and understanding similarities and differences across groups of terms within and between academics and young people. One researcher (RD: Psychology) completed line-by-line coding and preliminary codes were reviewed and agreed with another researcher (GP: S), and together they established preliminary themes (V1). V1 themes were reviewed and developed into V2 by two additional researchers (PC: AH; SHJ: S) before sense-checking by the YPAG in two separate sessions (see supplementary materials). Taking learning from these steps, RD and GP developed V3 themes for review by the wider interdisciplinary team involved in the CREATE project (see supplementary materials for themes, codes and quotes for each term).

## Results

We report important similarities and differences across academics’ and young people’s understanding of our selected terms. Our original groups (Research terms and Mental Health terms) were then divided into several themes. In the various phases of the project, discussion focused organically on 11 of the original 14 terms. Thus, we did not gather enough data to include the terms ‘Stigma’, ‘Empowerment’ and ‘Evidence’. Table 5 shows the relationship between the terms discussed and the themes that emerged.

**Table 5:** Themes derived from discussion of terms.

Group	Terms discussed	Themes
Research terms	Safe space	Generating vs Experiencing a Safe Space
	Connection, Community	Mutuality and Dynamics of Connection and Community
	Research, Data, Agency	What is research and why do it?
Mental Health Terms	Loneliness	Navigating the Complexity of Loneliness
	Mental health, Wellbeing,	Beyond the Diagnosis: Exploring Mental Health and Wellbeing
	Coping, Resilience	Unravelling Coping and its association with Resilience

### Group 1: research terms

Discussion of these terms (see Table 5) demonstrated notable differences in the way they were understood by participants. Moreover, these differences generated a good deal of surprise, as these terms where generally considered to be a ‘given’ by participants in the context of research.

#### Theme 1: generating vs experiencing a safe space

Trust was considered the key ingredient required to create ‘safe space’ by both young people and academics. Academics from across the disciplinary spectrum talked about safety in terms of their duty to create a trustworthy research process (practical and relational), whereas young people talked about a ‘safe space’ as a deeply personal, felt experience.

For many academics, ‘safe space’ was generally defined as a psychosocial context which must be created via a trust–building process and the showing of care and concern:

*you build kind of trust, and yes, some people sort of describe that synonymously with safe space, it’s safe, more in the sense that you first create that trust and that care*. (AH).

‘Safe space’ was also related by academics from a variety of disciplines to safeguarding / duty of care, and as a fundamental part of research culture. Efforts to create safety through trust were seen as especially critical for co-discovery mental health research that enables young people to talk about ‘*their self-harm or other sensitive topics*’(S). and in which ‘*your abilities are recognised, and your struggles are accommodated*’ *(*AH), One academic stressed that a ‘safe space’ does not mean ‘*that nothing [bad]* *happens*’ (C). This was also particularly emphasised by the Arts and Humanities academics involved:

*there’s an awful lot to talk about these projects as providing safe spaces, which in lots of ways is antithetical to the production of good art […] because quite often art is about rupturing safe spaces is about taking risks about pushing boundaries*. (AH).

This is a view, however, that is often seen to jar with institutional logics and practices, especially requirements to define (ethical) ways of working in advance. This was reported to be challenging for researchers who know the importance of being flexible and adaptive in building trust and safety when working with young people. Academic respondents frequently saw their goal as creating a research environment for young people that generated ‘*optimal allowance of activity*’ (AH).

Young people, conversely, were inclined to associate ‘safe space’ with community and interpersonal connection. It was seen as experiential, as a place where *‘you can get away from any stress or issues you have in your life’* or where *‘someone feels comfortable and calm to be themselves and be open […] or maybe even just a space where you feel happy’*. In this way, young people associated ‘safe space’ with feeling oneself and at ease, as ‘*somewhere you feel protected, calm and loved […] The sense of not knowing everything is okay when you are in that space’.* *It was also characterised as a place* ‘w*here you feel safe to talk about things and somewhere with people you know you can trust,*’ reflecting the opening-up that can be experienced once safety is felt. Thus, although academics and young people talked about different dimensions of ‘safe space’ these were not mutually exclusive.

#### Theme 2: mutuality and dynamics of connection and community

The term ‘connection’ was generally immediately oriented to by the academics involved as a facet of a good research context, referring to the need to build rapport with participants, and often characterised by ‘*shared understanding and shared goals’* (AH) or as ‘*feeling similar to someone’* (AH). This view was also partly held by the young people, but they mostly oriented to the term as an intensely personal, emotional connection in everyday relationships. It was described as a ‘*spark or fire’* that can evolve into a ‘*stronger bond*’, and as often being characterised by reciprocal care:

*it can be a feeling of closeness with someone. Caring for other people, looking out for them, wanting them to do the best that they can, and them feeling the same way about you, can show that you have a connection with them*.

While academics generally viewed ‘connection’ as necessary for successful research, young people did not consider it important in research, given their different orientation to the term.

Both groups felt ‘connection’ was central to the term ‘community’. Both felt that there ought to be some degree of commonality in, or belonging to, a group or community for there to be ‘connection’. One academic described ‘community’ as ‘*a group with coherence of purpose OR identity OR characteristic*’ (S). Similarly, one young person defined ‘community’ as *‘a group of people that come together as they have a similarity or commonality between each other. They come together and do activities together or share ideas’*. Notably, both academics and young people were wary of categorizing a community as a necessarily homogenous entity, pointing out that this can unhelpfully conceal the nuances of individual identity. One young person of Indian heritage, born in the UK, explained: ‘*I’m like in the UK and then I’ve got an Indian identity and how sometimes there’s a disconnect in the community because it’s like we’re not one or the other’*.

Some academics went on to even suggest that the term ‘community’ could be seen as dismissive, divisive even, being used to marginalise a particular group by presenting them as ‘other’:

*So the concept of a community [can be] a way of kind of ‘othering’ a particular group of people* [here the respondent was referring to the LGBTQ+ ‘community’] *and precisely not thinking about them as individuals, but sort of like […] a mass of people, that are undifferentiated, [even though] the experience of being within that community was often very, very different for different people*. (AH).

#### Theme 3: What is research and why do it?

One of the most surprising discussions was the differences in assumed understanding across the participants about what is meant by ‘research’ and ‘data’ and how they relate to the question of ‘agency’. Both academics and young people saw ‘research’ as ‘*a way of discovering new things and ideas*’ (YP) and a means of *‘looking to explore or find out more about something we don’t yet know*’ (S). However, young people presented a far stronger sense of power asymmetry and ‘othering’ in their responses, which conveyed a perceived lack of ownership of the research process. Young people talked about researchers using ‘*their words*’, ‘*their data*’ to position young people within a research project in a particular way that was not necessarily of the young person’s choosing and, in doing so, limiting their agency (a term discussed further below). Academics, particularly those working in the social and medical sciences, perceived that ‘research’ has ‘*an air of formality*’ that ‘*sits in certain contexts and paradigms*’ involving a ‘*process of structured enquiry*’ (C) that may, despite best intentions, create distance and inequity between academic researchers and participating young people.

The young people’s perception that research was often ‘done to’ them, despite efforts to create equitable and safe spaces, was illustrated well in the Central Living Lab task using music to communicate the terms ‘research’ and ‘data’. One group created a piece of music with a repetitive, ominous, threatening rhythm that communicated, for the young people involved, the sense of discomfort they associated with ‘research’. Another young person explained their music associations with the term in powerful ways:

*I think I’d have a very strong bass, like ‘dun’. I know you have […] a ticking clock but I’d have like something quite like dull or firm, even if it was just like a drum just going ‘dun, dun, dun*’ *in the background, just so it gives like a, I’m going to say hardcore, but you know like really bold like dark kind of thing*.

These ideas were extended by another young person in the group who wanted the composition to also reflect research as frenetic:

*maybe add like contrasting like melodies or harmonies and things like that to give it […] like a chaotic maybe kind of feel, something like that. I really like the way you structured it as well because you were going with research and research is frantic anyway*.

It should be clarified at this point that this was a purposeful discussion *in* the Living Lab, a space co-designed with young people (see supplementary material). Most young people’s understanding of research appeared limited to quantitative methods that, as one young person put it, involves ‘*numbers, statistics, […] giving them questionnaires, things like* *that*’. Some in the group were confused with the idea that art-based, creative methods could even constitute research:

*So when like we’re talking about like this [the music in the Living Lab] as research, I was like ‘well this is not the research that I know* *of, but, you know, I was confused. How are you measuring whatever we’re doing if you know what I mean?*

This was also a particular fault line across the academics involved in the study. While, for medical and social science academics, research was delimited to ‘set activities, set processes’ that were, crucially, ‘replicable ’, for some Arts and Humanities academics, research was seen as a more open process:

*certain colleagues […] particularly the more scientifically minded have a real clarity about what research was and what it wasn’t […] whereas those of us more focused on co-production, using the arts, there is more fluidity between ‘intervention’ and ‘research’*.

For the Arts colleagues involved, this related to the fundamental question of what the ‘point’ of research is, which was frequently seen to be about opening up new avenues of enquiry, or generating new ways of looking at a given topic, as opposed to finding specific ‘answers’ to a given topic. This was further reinforced in our discussion of ‘data’. One social scientist reflected on her recent experience with the ABP of participatory video as a research method, stating:

*Actually, it’s interesting to see […] how we all define what data is and I think particularly makes me think you know how data can be anything in a way […] It’s really challenged my belief of what data is and what it actually looks like* (S).

Another highlighted the transformative potential of arts-based data, noting what they saw as its ability to unearth insights not accessible through traditional scientific methods. The approach to data creation taken during the Central Living Lab was seen as challenging ‘*things that are taken for granted in science*’ and may ‘*flip the narrative*’ (S), unearthing insights not accessible through traditional methods and thereby offering opportunities for engagement and communication appropriate for diverse groups of researchers/participants, allowing all groups to contribute to the project on their own terms, and to have their contribution valued. This was raised by some participants in the study who identify as neurodivergent as being particularly helpful. However, conversely, this sense of openness seemed to be at odds to the very purpose of research for other participants. If the purpose of ‘data’ is to generate more new questions *‘it has limited value […] Data is that which provides information about the veracity of a specific statement…the less speculative the better*’ (C).

‘Agency’ was a term valued by academics from all disciplines and was deemed important in leveraging meaningful participation in mental-health research:

*I’m bringing an art perspective to it, but from my experience agency is one of the most important words I associate with mental health or doing mental health research because it’s about choice and control* (AH).

Most young people were unsure what ‘agency’ meant in relation to research and mental health and defined it as ‘*an organisation that offers a service*’. However, in the Living Lab, some of the young people experienced in research participation explained the term to other participants. Their explanation corresponded with how academics understood the term, with one young person defining it as, ‘*Having control over your actions and the way you live your life*’.

### Group 2: mental health terms

There were more points of correspondence in the ways in which young people and academics understood the mental health terms discussed (see Table 5).

#### Theme 1: navigating the complexity of loneliness

Both academics and young people associated loneliness with a lack of belonging or companionship (YP: ‘*feeling like you don’t belong somewhere - feeling left out from a community or a group of people*’*;* S: *‘feeling disconnected and having a sense of being on the outside*’). One young person’s description offered a more nuanced and multifaceted perspective:

*it definitely brings out a lot of emotion and explores the more like tragic and painful sides to loneliness, especially someone who’s experienced and been through a lot of loneliness for a long period of time*.

Young people’s accounts conveyed that loneliness is not simply a sense of disconnection, but often involves a long period of confusion, pain and complex feelings. The length of time one spends with feelings of loneliness seems to compound the experience and make it even more *‘tragic and painful*’. Most young people perceived loneliness as a very common experience and actually found solace in its commonality, a point also frequently made in the wider literature (e.g. Hawkley, Cacioppo 2010)): *‘like I’m human and everybody’s a human and everyone’s alone, so it’s quite* *empowering’*.

The young person linked loneliness with self-awareness, suggesting they associate the term with personal growth. Academics also associated ‘loneliness’ with normative experiences but noted their own distance from the phenomenology for young people:

*We understand it in psychology as partly an outcome of that developmental phase of finding your place in society, but I’m not sure that that’s a good enough explanation actually for what young people feel* (S).

The complex phenomenology of ‘loneliness’ was shared by one young person in their choice of music:

*[In this task] I chose [the song] Awakening by Ichika Nito and I linked it to loneliness and I felt […] like it linked to loneliness because of the contrasting harmonies in the song. The whole song seems and feels quite chaotic*.

Another young person also wanted to express how they associated ‘loneliness’ with a complex phenomenology:

*So in the lyrics, the singer talks about how […] the only friends he has are the voices in his head and how he’s effectively alone and no-one really understands what he’s going through and from the singer’s point of view it kind of describes his struggles with mental health*.

The sense of loneliness being seen as a burden was also reflected in other words academics and young people used to describe the experience. These included ‘*sad*’, ‘*depressed*’, ‘*helpless*’ and ‘*ominous*’.

#### Theme 2: beyond the diagnosis: exploring mental health and wellbeing

Young people held an understanding of ‘mental health’ that was generally synonymous with ‘wellbeing’. Academics perspectives were strongly rooted in their professional knowledge, which emphasised differences between the terms, and from different disciplinary perspectives.

While one academic defined ‘mental health’ as the ‘*absence of mental illness*’ (S), another contested this, viewing it as a ‘*scale of both positive and negative mental health*’ (AH). In this sense, mental health was viewed as similar to physical health, which can be positive or negative. An interesting discussion took place among the academics comparing ‘mental health’ with ‘wellbeing’. While ‘mental health’ ‘*has a very strong clinical connotation*’ (C), ‘wellbeing’ was often seen as ‘*overall mental health, holistically, taking account of social, familial, cultural contexts that contribute to wellbeing*’ (S). In this sense, ‘wellbeing’ was perceived as a ‘*nebulous concept*’, one that can mean ‘*different things to different people in a way that perhaps mental health doesn’t*’ (C). One academic focussed on the etymology of both terms:

*mental health has emerged from psychiatry, whereas wellbeing has probably more emerged from positive psychology and the idea is that we should be looking at not just what predicts disease, but also what predicts flourishing and doing well and having a good life* (S).

This suggests that ‘mental health’ is seen as disorder-focused, with an emphasis on prevention and treating clinical conditions, whereas ‘wellbeing’ relates to maintaining a positive mental state irrespective of the challenges faced by an individual. One academic explained this with an example:

*maybe […] I have a condition, a lifelong condition, that doesn’t have a cure […] and that might be synonymous with my mental health. […] and if I’m managing it and doing well in the world, then you could say my wellbeing is good. But my mental health in that context is [non-existent]* (AH).

The word ‘condition’ is linked to mental health, whereas ‘wellbeing’ is seen to involve managing ourselves despite adversities. For the academics, the terms were not synonymous, with wellbeing generally being seen as more ‘palatable’, and so more broadly useful – particularly within the context of ABP and particularly community-based participatory arts practices – than ‘mental health’: *‘Wellbeing is often much more* “*connected*”. *And to do with actions and activities and much more situated in the world*’ (S). Or as one Arts academic put it:

*I wonder if the wellbeing side of kind of community arts is much more about kind of people keeping fit and being healthy and creating community and all that kind of stuff. Whereas, when people talk about mental health and arts is that much more kind of music therapy, art therapy, […] specifically kind of a clinical practice in terms of the art* (AH).

Although academics generally described ‘mental health’ and ‘wellbeing’ in psychological terms, one young person associated ‘wellbeing’ with both the psychological *and* the physical: *‘a combination of physical and mental comfort, both of which needing to be positive to have a positive wellbeing*’ (YP).

Overall, compared to academics, young people offered a more straightforward understanding of ‘mental health’, albeit one that often fused with ‘wellbeing’. One young person said that ‘mental health’ is ‘*how well a person can/is able to deal with certain things they go* *through’*. This description is closer to academics’ description of ‘wellbeing’. One young person defined mental health as having an underpinning biology which we need to understand if one is going to do something practical to improve it:

*How healthy your mental state is. This can be more subjective in terms of how someone feels but also biological in terms of diagnosing someone with a mental health condition*.

Apart from this young person, no other academic or young person drew upon any clinical vocabularies when explaining their view of the term ‘mental health’, despite defining it at times as a ‘clinical condition’. Young people described the term ‘wellbeing’ in a similar way to the academics. For instance, in choosing a song to represent wellbeing, one young person suggested:

*I chose Forget You by CeeLo Green because it implies wellbeing, because he’s putting away the pains of his past and he’s moving forward in a positive and happy way and overall the song is just really nice and happy*.

Interestingly, this account of wellbeing also connects with discussion of the term ‘resilience’ (see following section), suggesting that ‘*moving forward*’ from pain can lead to maintaining wellbeing and feeling ‘*positive and happy*’ (YP).

#### Theme 3: unravelling coping and its association with resilience

There were similarities in the way young people and academics viewed the term ‘coping’ but differed in their view of the term ‘resilience’, especially with regard to its perceived value to young people. In discussions of ‘coping’, both young people and academics saw it as a form of self-regulation aimed at concealing problems. One young person described coping as ‘*dealing with negative emotions by suppressing them and refusing to outwardly share them*’. This association was echoed by an academic (S) who likened ‘coping’ to ‘*camouflaging*’ of true experience. Some academics expressed concerns that the use of the term ‘coping’ might perpetuate a cycle of avoidance or denial, holding individuals back from addressing underlying issues: ‘*I might be coping because I’m not screaming now, because I’m somehow managing not to scream. But does that mean I’m OK?*’ (AH).

The term ‘resilience’ elicited various points of view among academics. One considered ‘resilience’ as a form of resistance to unreasonable demands:

*I just think […] being resilient is like being able to kind of safeguard yourself in terms of that you can like, resist or challenge […] if demands are unreasonable or situations unreasonable* (S).

Other academics raised concerns about the potentially careless use of the term that one might find in non-psychology-led research, such as the kinds of ABP research being used across CREATE (as issue raised, however, by an academic coming from outside of Psychology):

*Resilience is a way of teaching people to put up with situations that they shouldn’t put up with. And I wonder in terms of arts-based mental health research, […] how much is a focus on resilience actually a problem, as opposed to something that we should be instilling in people* (AH).

Here there is an issue raised about an overuse and emphasis of the term ‘resilience’ that could overlook the adverse conditions impacting an individual’s mental health, thereby undermining efforts to address systemic barriers to wellbeing. No consensus emerged among academics on this term, as there were some who perceived ‘resilience’ to be something that ‘*strips people off completely from hope and self-esteem*’(S), while others saw its value as a safeguarding mechanism, helping people ‘*get through things*’ (C).

In contrast, young people only saw value in having an ability to be resilient:

*the word resilience to me means to continue going on in life because there are going to be moments in life [when] you feel hopeless, [when] you feel like nothing’s going to get better but at the end […] you’re going to look back and you’re going to feel happy that you’ve gone through it*.

Young people viewed the term as being cognate with phrases such as ‘*adapting and changing*’, ‘*letting go*’, ‘*becoming stronger*’ and ‘*being able to bounce back from a more negative situation*’.

## Discussion

Adolescent mental health research remains a global priority. Given the present emphasis on participatory and interdisciplinary ways of working, we need to do more to develop shared understandings of vocabularies and values stemming from the different disciplines that contribute to youth mental health research, as well as the lived experience of young people, if we are to practice equitable and inclusive research in this space. This study aimed to contribute to this need by examining some of the key terms in youth mental health research, as expressed by a diverse group of young people and academics from different demographic and disciplinary backgrounds. In so doing we sought to position all forms of knowledge as equal. Our study drew from the social sciences, arts, clinical, youth and lived experience perspectives. It is not our aim to create a single, agreed meaning for each of our terms, but rather to understand differences in disciplinary understandings, ultimately with the aim of working with these differences, indeed understanding and exploring the dynamics at work between them, in order to improve mental health research with young people (Jennings et al., 2006).

As one might expect, overall, the academics’ understanding of the terms was rooted in their disciplinary homes while the youth co-researchers generally foregrounded the link to their lived experience. With regard to ‘safe space’, for example, although trust was considered a cornerstone of a safe space for young people and academics, academics focussed more on institutional and procedural trust and young people on relational and emotional trust. Academics’ perception of ‘safe space’ at times reflects broader principles in research ethics, where dependability, honesty, and the commitment to minimizing harm are paramount (Hugman et al., 2011). That said, academics, particularly those working in the arts and humanities, lobbied for more openness and experimentation in what institutions are able to conceive of as ‘safe’. Young people associated ‘safe space’ more with emotional security, interpersonal comfort, and community. As mental health research often involves sensitive and emotionally charged topics (Pinto et al., 2022), there is a need for academics to reconcile these differing expectations of ‘safe space’ to accommodate trustworthy research processes that can foster innovation and experimentation, and on the other hand, the emotional needs of participants. While academic discourse often emphasizes ethical and procedural frameworks for safety, young people describe safe spaces in deeply experiential and relational terms—places where they feel protected, accepted, and emotionally secure. Such insights point to the need for a broader, more youth-informed definition of safe space—one that incorporates the affective, social, and contextual elements young people themselves prioritize.

Similarly, our findings show that young people understood ‘connection’ as emotionally rich and grounded in mutual care and evolving relationships. In contrast, academics framed ‘connection’ more instrumentally, aligning it with rapport and shared goals within a research setting (in a similar way to e.g., Bell et al., 2016). This divergence likely reflects differing orientations—young people draw from everyday lived experience, while academics work within disciplinary norms. As such, researchers must use terms like ‘safe space’ and ‘connection’ carefully, ensuring they reflect young people’s perspectives. Young participants emphasized that reciprocal relationships are essential to feeling valued and contributing meaningfully to research. Yet, this emphasis often clashes with the fast-paced nature of academic research and PPI (Patient and Public Involvement), which tends to prioritise outputs over deep relational work (Escobar, 2011). Bakermans-Kranenburg and van IJzendoorn (2024) caution that time constraints in academia can hinder the development of trust in PPI contexts. Likewise, Abebe (2019) underscores the ethical necessity of reciprocity and emotional safety in participatory work with young people. Our findings build on this by showing that connection is not just a method or outcome, but a baseline condition for meaningful, youth-centred engagement.

Moreover, we can see the power asymmetries uncovered in the way young people understood fundamental terms such as ‘research’ and ‘data’. This emphasizes the need to create a greater sense of trust and reciprocity in order to develop a sense of equity amongst different stakeholders. This can help participants, in this case young people, to feel a greater sense of ownership of the research process. With regard to research and data, it was also noticeable that this issue is compounded by the fact that there are conflicting understandings of what these terms mean amongst academics. Participants from arts and humanities often saw research as a way of provoking new questions that can generate data open to interpretation, while those from clinical and social science backgrounds saw open interpretation of data detrimental to research processes that should *answer* and not *generate* questions.

Turning now to our mental-health-related terms, young people’s conceptualisation of loneliness emphasises the multifaceted nature of this experience where, for some, the concept actually provided a commonality across the group. For others, the concept of loneliness pointed to a lack of understanding of one’s own ‘chaotic’ inner feelings. Such perspectives challenge conventional portrayals of loneliness as a passive state (Van Buskirk and Duke, 1991), highlighting how it can both impact, and be actively operationalised by, young people. Recent work by Qualter et al. (2023) similarly conceptualises adolescent loneliness as a dynamic and contextually driven experience, rather than a static or purely internal condition, supporting our observation of its complex and expressive character. Importantly, these perspectives emphasize the value of hearing directly from young people about their experiences of loneliness, particularly in relation to feelings of belonging and identity. This resonates with Garcia et al. (2023), who found that a strong sense of community acts as a protective factor against loneliness and supports better mental health outcomes among adolescents. Intersectional factors such as age, ethnicity, disability, and other social identities play a crucial role in shaping how individuals experience loneliness and navigate feelings of connection (Yang, 2023), as is also apparent in our young participants’ discussion of ‘community’. Smith et al. (2023) further argue that overlapping marginalised identities, such as being LGBTQ+ and from an ethnic minoritized group, can intensify feelings of isolation, underscoring the need for intersectional frameworks in loneliness research. Understanding how these layers of meaning interact can broaden mental health research and interventions, moving beyond a narrow focus on loneliness as merely a symptom of social isolation to a more nuanced understanding that includes the role of belonging and intersectionality in these experiences (Jenkins, 2023).

The theme of mental health and wellbeing revealed interesting differences in perceptions between young people and academics. The young participants often viewed mental health in broad, subjective terms, frequently intertwining it with the concept of wellbeing. On the other hand, academics tended to distinguish between mental health and wellbeing, rooting their understanding (at least implicitly) in clinical and historical contexts. This distinction is consistent with the wider literature, which has historically treated mental health as the absence of mental illness, often aligning it with clinical diagnoses and biomedical models (Zannas et al., 2023). Young people’s more integrated view of mental health and wellbeing suggests that these concepts are not experienced as distinct in their everyday lives. For young people, mental health is less about the presence or absence of illness and more about the ongoing ability to cope with life’s challenges, an idea that closely aligns with the way they understood both wellbeing and resilience. This resonates with the WHO’s own evolving definition of mental health as not just the absence of illness, but a state of wellbeing in which individuals realize their potential, cope with stresses, and contribute to their communities (WHO, 2022). This calls, perhaps, for more exploration of comprehensive mental health strategies that incorporate both preventative measures and the promotion of overall wellbeing. Our findings underscore the need for these strategies to reflect the lived experiences of young people, emphasizing subjective meaning-making, emotional depth, and relational factors—dimensions often underrepresented in traditional academic approaches (Zannas et al., 2023).

Notably, when one academic (S) discussed mental health in terms of the absence of illness, it was vigorously contested by others (AH and S), who perceived it as an ever-existing state that can be positive or negative. The shift from using the term ‘mental illness’ to ‘mental health’ to reduce stigma is a crucial point in this regard (Schomerus et al., 2016), but it also brings challenges. By framing mental health in broad, positive terms, there is a risk of obscuring the experiences of those with severe mental health conditions (Herrman, 2001; Schomerus et al., 2016). Therefore, it is essential to strike a balance in discourse and practice: while promoting mental health and wellbeing for all, there must also be a continued focus on the needs of those with profound mental health challenges (Herrman, 2001). When conceptualizing mental health in research, it is vital to involve ‘experts by experience’ early in the process. By involving YP at the outset of a study, academics can be guided on dimensions of mental health that are most relevant. This ensures that research addresses real-life implications and fosters practical outcomes that truly benefit those affected.

The discussions around coping reveal its dual nature as both a necessary survival mechanism and a potential barrier to true emotional processing (Butler et al., 2003). Both young people and academics recognized coping as a form of self-regulation, often involving the suppression, or concealment, of emotions. This aligns with psychological theories that view coping as a strategy to manage stress and maintain functioning (Stephenson et al., 2016). However, the concern expressed by academics that coping may prevent individuals from addressing underlying issues raises important questions about its long-term efficacy. This suggests that coping, while useful in the short term, may contribute to chronic emotional suppression, which could exacerbate mental health issues over time (Geraerts et al., 2006).

The debates around resilience among academic participants touch on a significant issue: encouraging people to be more resilient might inadvertently support a neo-liberal agenda that emphasizes self-reliance over collective care and support (Spolander et al., 2014). This perspective argues that resilience, when promoted without a critical examination of the broader context, can lead to the neglect of systemic issues that contribute to adversity, such as inequality, discrimination, and lack of access to resources. A dynamic approach recognizes that mental health is not just about individual coping mechanisms but also about the capacity to engage with and adapt to changing circumstances, often requiring external support (Ungar, 2011). On the other hand, young people overwhelmingly viewed resilience as a positive trait, one that allows them to ‘bounce back’ from challenges and continue moving forward. This suggests that resilience, for young people, is closely linked to a sense of empowerment and self-efficacy. The divergence we see in perspectives between young people and academics problematizes the use of the word resilience in research. The findings suggest that mental health strategies should carefully balance the promotion of resilience with efforts to address the root causes of distress.

## Limitations

This study had limited co-analysis with young people. While member-checking was used, deeper involvement in data interpretation could have strengthened youth-led insights. The research team’s demographic—predominantly white, middle-class academics—may have shaped the framing and interpretation of findings. Group-based focus methods, even with anonymous tools like Padlet, may have limited some participants’ expression. While the study sought to look for broad trends in the way terms were used by different people across our research team, one must be cautious in drawing generalisable conclusions from it. Perspectives may differ across neurodivergent youth, global majority groups, and varied socioeconomic backgrounds. Concepts like ‘resilience’ may be defined differently across cultural and contextual settings (Ungar et al., 2005). Finally, the relatively short duration of youth engagement may have constrained the depth of contributions; longer-term collaboration could yield richer insight.

## Conclusion: toward a new model—the EQUITY framework

Creating opportunities for open communication and shared understanding are crucial if youth mental health researchers are to build the necessary foundation of trust and mutual respect to work effectively with young people. In the process this can help to demystify the research process for young people and promote a culture of co-creation and collaboration that can meet the expectations of all stakeholders. In such an environment, young people are more likely to feel empowered and engaged, contributing to research that is both meaningful and impactful. Moreover, by generating equitable, non-hierarchical relationships in a study that seeks to respect and engage knowledge and perspectives of other groups and generate dynamic encounters between these groups, gives academics the opportunity to gain new, unexpected insights. This approach underscores the importance of incorporating diverse perspectives and experiences into research that can ultimately lead to richer, more reliable, findings (Jennings et al., 2006). It also, crucially, emphasises the fact that interdisciplinary dialogue of any type cannot be rushed, especially when working with young people. Surfacing the insights that can emerge from these kinds of encounters needs space for mutual trust to emerge, trust that takes time to develop and can also be lost in an instant if young people think that such engagement is perfunctory and that their contribution to the project is not genuinely valued and respected.

**Fig. 2:**
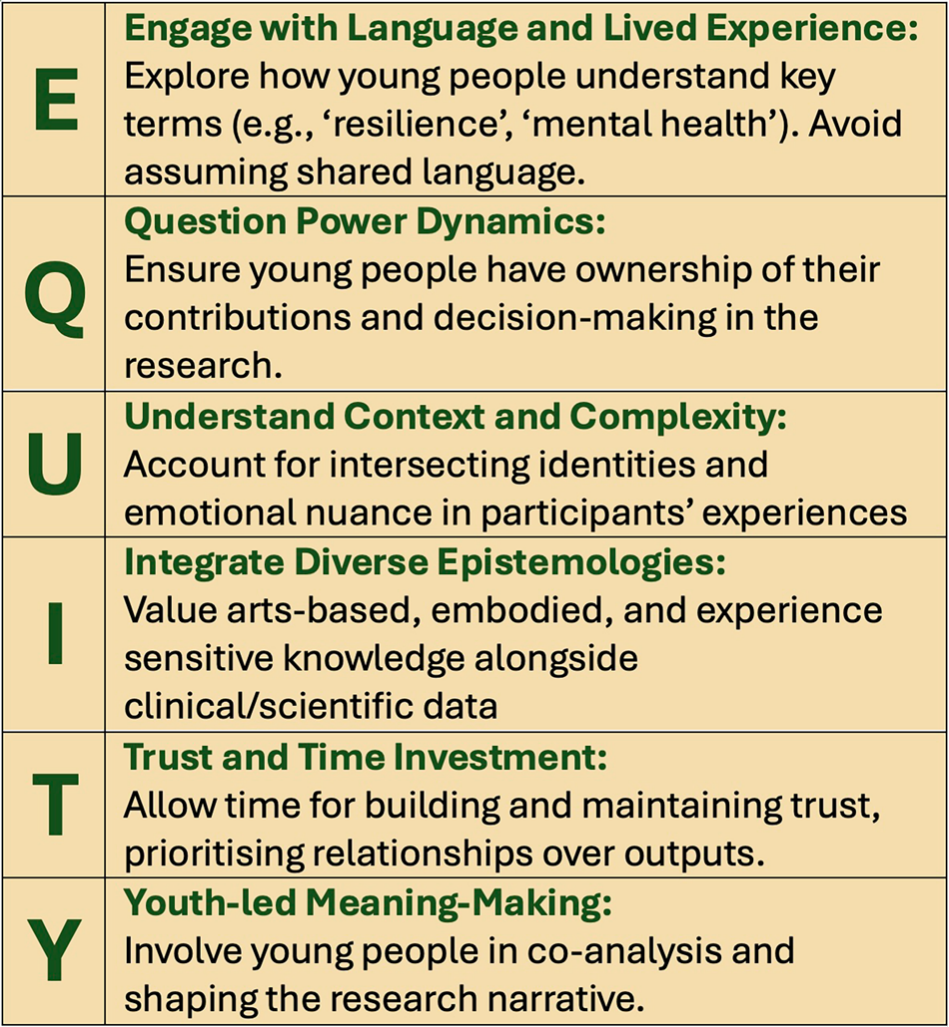
Visual representation of the EQUITY framework, a practical guide to inform inclusive, youth centered mental health research.

Our research adds depth to the field (eg Woodgate et al., 2023) by examining not only *what* should be done to support meaningful youth participation, but *how and why* these approaches matter in practice. Based on the insights from this study, we propose the EQUITY Framework as a practical guide to inform inclusive, youth-centered mental health research. It brings together the key themes uncovered—such as power, trust, language, emotional safety, and participatory ownership—into an actionable model for both researchers and youth participants (see Fig. 2). This tool is designed to help researchers embed inclusive, trust-based, and meaningful practices throughout the research process:

The *Checklist for Researchers* translates the six EQUITY principles into reflective prompts that encourage self-awareness and accountability (Table 6). These questions guide research teams in examining how power, identity, language, and epistemology shape their practices and relationships with young people. They are intended to inform study design, implementation, and dissemination phases alike.

**Table 6:** Checklist for Researchers Using the EQUITY Framework.

Principle	Key Practice Questions/Prompts
**Engage with language**	Have we explored how young people understand key terms? Do we assume shared language too quickly?
**Question power dynamics**	Do young people have ownership over how their contributions are represented?
**Understand context and complexity**	Have we acknowledged intersecting identities (e.g., ethnicity, neurodivergence)?
**Integrate diverse epistemologies**	Are arts-based and experiential knowledge treated as equally valid?
**Trust and time investment**	Have we built enough time into our project for genuine relationships?
**Youth-led meaning-making**	Are young people involved in shaping the ideas or conclusions of the research?

The **Checklist for Young People** offers a parallel tool, written in accessible language, to support meaningful youth participation (Table 7). It provides young participants with questions they can ask themselves or the research team to ensure their voices are respected and their involvement is not tokenistic. This checklist affirms young people’s right to understand, challenge, and co-direct the research they are part of.

**Table 7:** Checklist for Young People using the EQUITY Framework.

EQUITY Principle	Questions You Can Ask
**Engage with Language**	Do I understand the words researchers are using? Can I ask for things to be explained or say what makes more sense to me?
**Question Power Dynamics**	Do I feel heard and respected? Can I help shape how my ideas and stories are used? Can I say no without feeling bad?
**Understand Context and Complexity**	Do the researchers understand my background, culture, or identity? Do I feel safe being myself here?
**Integrate Diverse Epistemologies**	Can I share ideas through art, music, or other creative ways? Is my experience seen as important, even if it’s not ‘data’?
**Trust and Time Investment**	Have we had enough time to build trust? Do I feel comfortable asking questions or sharing my thoughts?
**Youth-led Meaning-Making**	Am I helping shape the project or the findings? Do I have a say in what this research is really about?

### Practical recommendations

The EQUITY checklists reflect a commitment to equitable practice grounded in co-production, mutual learning, and respect for diverse forms of knowledge. We recommend their use across youth mental health research projects, particularly those engaging with complex identities, trauma histories, or systemic exclusion. When embedded intentionally, they have the potential to move research beyond extractive models and toward more just and collaborative futures. In youth mental health research, the EQUITY checklists can be brought to life through intentional, practical actions. For instance, researchers can co-create a glossary with young participants to define key terms like “resilience” or “trauma”, ensuring shared understanding from the outset. To address power dynamics, they might invite youth to co-author papers or co-present findings (Sonuga-Barke et al, 2024; Pavlopoulou et al, 2022) and give final say on how their stories are quoted or interpreted. Understanding context means going beyond surface-level demographics, which can be done by using tools like identity mapping workshops to explore intersecting experiences such as ethnicity, gender, or neurodivergence. Embracing diverse ways of knowing could involve accepting drawings, music, or storytelling as valid research contributions, not just written or verbal responses. As mentioned earlier, trust-building requires time, and researchers might organize informal check-ins or social events outside of formal data collection to foster genuine relationships. Meanwhile, young people can advocate for themselves by asking for plain language explanations, challenging unclear terms, or expressing when something does not feel right. These practices ensure youth voices are respected and central and not tokenized throughout the research journey.

## Supplementary information

Supplementary Materials

## Data Availability

Relevant data is provided in Supplementary materials S5 that include anonymised quotes, codes and descriptive themes. Individual anonymised transcripts can be made available on request.
