# Supplementary material for: Exploring similarities and differences in how researchers and young people understand key terms in youth mental-health research
Source: Humanit Soc Sci Commun. 2025 Sep 29;12(1):1521. doi: 10.1057/s41599-025-05809-5 (PMC12479358; doi:10.1057/s41599-025-05809-5)
Supplement: Supplementary file 1 — Supplementary Materials [file 41599_2025_5809_MOESM1_ESM.pdf]

Supplementary Table S1

Demographics for the Delphi survey

Team	Members	Self-description of role in mental health research projects	Department
CREATE team members	D1	Researcher with expertise in child development and qualitative research	Faculty of Health and Medical Sciences, Psychology
	D2	As a (social scientist) psychologist viewing mental health from both realist and constructionist perspectives, and the role of culture, politics and power in shaping MH experiences – ie it is more than an individual's problem	School of Psychology
	D3	I contribute expertise in socially engaged creative practice (applied theatre and performance and multi modal arts-based practices), creative research methods and neurodiversity.	School of Arts
	D4	An arts practitioner and a humanities/interdisciplinary researcher	School of Languages, Cultures and Societies
	D5	I am a musician and music psychologist with experience in qualitative methodologies, particularly interpretative phenomenological analysis (IPA). I have specialised in uses and subjective experience of music in everyday life, including a phenomenological study of young people's experiences (aged 10-18).	Dept Music & Audio Technology
	D6	Associate Professor Arts and Health	Centre for Arts and Health
	D7	Researcher	Child & Adolescent Psychiatry
	D8	Methodologist (CBPR, quals, mixed methods) with particular interest and experience in coproducing and codelivering with YP	Brain Sciences
ATTUNE project team members	D9	Researcher	Not provided
	D10	Creative and technical collaborator	Games Academy
	D11	Autistic Post-Doctoral researcher	School of Arts
	D12	Not provided	Not provided
	D13	Co-ordinator and facilitator	School of Film and Television

	D14	I am there to ensure the Research is conducted in proper manner, fairly, ethically and adds value to the target end user/stakeholder.	Games Academy
	D15	To facilitate a creative space where participants can explore and express themselves in a safe space.	Institute of Photography
	D16	Researcher	Wolfson Institute of Population Health/ Faculty of Medicine and Dentistry
Others	D17	Research Assistant	Psychological and Social Medicine
	D18	Lecturer in the Psychology of Childhood and Education	School of Education
	D19	Firstly a Clinician and then an academic, both of which focus on the wellbeing of young people and how to progress the conversation and thinking in this area.	Leeds Institute of Health Science, School of Medicine
	D20	I am programme manager on a mental health research project. I aim to support and facilitate the research processes and particularly the communication and collaboration between the different groups in our research community, eg investigators, researchers, youth researcher panel, collaborating charities, schools, NHS and other organisations.	Not provided
	D21	I am a Research Assistant in the COMIC research team. We develop research ideas in collaboration with children, young people and their families, clinical staff, and academics with a vested interest in the improvement of mental wellbeing in children and young people.	Child Orientated Mental Health Innovation Collaborative (COMIC) Research Team
	D22	Director<br>The work we do supports people, young people, adults and older adults, in improving their mental health and wellbeing, wherever they are on the spectrum of mental health. We do this from a background of understanding the broad range of mental health need, the services currently available and the vital role that creativity can play in this area.	Art for Health Cornwall
	D23	Researcher in youth mental health	Health in Social Science

	D24	I am a consultant and researcher working with emerging adults	Academic Psychiatry, Psychological Medicine
	D25	Researcher who has focused on the coproduction of wellbeing surveys for children in care (4-18yrs) and care leavers (18-25yrs) AND using the findings to work with local authorities to change policy and practice	Department of Education
	D26	Professor of Applied Social Science	School of Education and Social Work
	D27	Experienced qualified youth worker supporting young people in marginalised groups for example LGBT, disability, young carers, adopted. Mental health and suicide prevention trained; some specific training for example eating disorders.	Not provided
	D28	Software Developer - Creating software designed to improve mental well-being using data and evidence provided by experts in the mental health field and in collaboration with young people to ensure it meets their needs.	Not provided

Supplementary Material S2

Listening Lab Protocol
Venue: Eiger Studios, Fairfield House, New Craven Gate, Leeds LS11 5NF

Participants: 20 young people, 11 aged 10-17 and 9 aged 18-24
Sessions recorded via video cameras in the room and via notes on the central project flip chart

Day One
Session 1 (90mins):

- Young people all come into central space and sit on benches together. 2 Music Facilitators (F1= Jackie, F2= Paul) and 4 members of the project team join the group and introduce themselves.
- Group introductions and sharing aims of the Listening Lab with the young people.
- F1 leads an icebreaker: 'if you were a piece of music, what music would you be?' discussion.
- Project team co-produced a 'charter' for the Lab:
    - What do we want to get out of this weekend?
    - How should we treat each other?
    - Agreeing that all activities are an 'offer' and there is no expectation that anyone *had to* take part.
- F2 leads an introductory session. 14 terms were written in post-it notes and placed in front of the room and young people were asked:
    - how do the terms we are looking at make you feel and why?
    - If the terms were sounds, what sounds would they be?
- Young people chose a space in the studio to record themselves explaining how their chosen song/music connects to their selected term

Session 2 (90 mins): Introduction to sound and rhythm.

- Young people reconvene in the central space.
- In groups (as before) they discuss:
    - If you were an instrument, what would you be and why?
    - Groups share what music they listen to, when, where, and why? (e.g. for changing mood, escaping, zoning out, studying, coping, creating a private space, creating energy.
    - They think of a time when they are listening to music, describing what they notice, what they're doing, how they feel.
- F1 leads a series of exercises getting participants to copy clapping/stamping rhythms. The rhythms get gradually more complicated and increasing in number involving the young people improvising.
- F2 introduces the group to the Band Lab app, encouraging participants to play with the app to make their own rhythms.
- Session ends with group listening to each other's rhythms.

Session 3: (90 mins): Sonic Islands and Private Spaces (Alone v. Lonely)

- Participants work independently to create an imaginary island where they either feel alone or lonely. They are asked to respond to a series of questions to help them build their island.
    - Where is their island?
    - Where do they live on their island?
    - What can they see?
    - What can they hear?
    - What's the weather like?
    - How do they feel about living there?
    - What would they like to change?
- Participants get into pairs to discuss their islands.
    - Using the Band lab app, each pair works together to create a piece of music that captures their island in sound/music.
    - The group comes back together to listen to everyone's work and discuss what they think about the music.
        - How does it make them feel?
        - Is this what the producers of the music wanted the listener to feel?

Session 4 (30 mins): Working with our terms

- Participants go into their groups of 4
- Each group is asked to cluster all the terms into groups of 3-4 terms that they think are similar to each other.
- Each group presents their thoughts to the rest of the group about why they have grouped the terms as they have.

Session 5 (30 mins): Group feedback on the day

- Participants fill in a very short questionnaire on the day's activities and what they might like to do differently on day 2.

Day Two

Session 1 (90 mins): Recap and warm up (Data captured via video and sound recordings in individual rooms)

- The whole group comes together to discuss feedback from Day 1
- F1 revisits the rhythm exercises from Day 1, but this time using musical instruments
- Participants go back into their groups of 4. Each group moves to a separate room, each of which has been set up with a random selection of musical instruments, including drums, guitars, pianos, keyboards, and lots of percussion instruments.
- Groups each revisit their sonic islands and create music that they think reflects what they wished to communicate via Band Lab, but this time with live instruments.
- F1 and F2 visit each group and listen to their music and discuss what they are trying to communicate.

Session 2 (90 mins): Refining the groups of terms and turning them into music

- Each group's grouping of the terms from Day 1 are written up on post-its and attached to the flip chart.
- Participants join together to decide on a final grouping of terms that they would like to explore for the rest of the day through music.
- Each group returns to their individual room and begins to explore what sort of music they think best fits their terms, using a version of the rhythm exercise they had been taught the day before.
    - One person starts a rhythm.
    - A second person adds to it to make it more complex.
    - A third person changes it further.
    - The fourth person changes it again.
    - The pattern is repeated until one of the groups feel that they have got to the end of this rhythm and they stop.
    - The group then reflects on what they liked about their music, what they feel they would like to do differently to better capture how their terms make them feel.
    - F1 and F2 and the project team circulate around the groups, offering reflections and support as required.

Session 3 (90 mins): Developing their sonic responses to their terms

- Remaining in their individual rooms, the groups continue to work on their music, deciding how best to communicate their terms using sound.
- Each group also discussed what they were aiming for.

Session 4 (90 mins): Final Concert and Discussion

- Participants come together in the main hall.
- Each group in turn plays their musical piece.
- The other groups attempt to guess which terms were the impetus for this piece of music. This is followed by a further group discussion about the music and what it says about the terms.
- The session ended with a group discussion about the living lab and how they feel the two days have worked.

Supplementary Material S3

Regional workshop (23rd September 2023, 10am-5pm)
Venue: The House Youth Centre, Carlyon Road, St. Austell, PL25 4BX

Participants: 10 young people, aged 11-15
Sessions recorded via video cameras in the room

Introduction Session (10-11am):

- Young people all come into central space and sit together. Breakfast provided.
- 2 Music Facilitators Giles (GW) and Pema Wainwright, 2 members of the project team and 1 member from Artswell joined the group and introduced themselves.
- Group introductions including YP sharing their musical interests and the team introduced aims of the workshop to the young people.
- F3 leads icebreakers:
    - YP design their own name tags
    - Played 'Splat' as a group
- F3 along with the project team co-produced a plan for the workshop:
    - What do we want to get out of this weekend?
    - How should we treat each other?
    - Agreeing that all activities are an 'offer' and there is no expectation that anyone *had to* take part.

Session 1 (11am-12:30pm): Term grouping

- Project team showed YP the performances created during the first Living Lab, as an example of how they had interpreted the grouped terms through music.
- F3 leads the session. 14 terms were written in post-it notes and placed in a chart and young people were asked:
    - how do the terms we are looking at make you feel and why?
    - If the terms were to be grouped together, what would they look like?
- Young people organised the terms into groups while discussing among themselves
- F3 invited YP to form groups based on the family of terms
- Each group provided with a plain sheet of A3 in which they were invited to express meanings of the terms using words or drawings

LUNCH BREAK (12:30-1:30pm)

Session 2 (1:30-4pm): Creating music

- Young people reconvene in the central space.
- F3 instructed YP to remain in their small groups based on the family of terms
- YP were invited to choose a space within the 3 rooms within the premises (leaving out the 'calm space') and started creating a musical piece reflecting respective family of terms
- Musical instruments included 2 drum sets, 1 electric guitar, 1 acoustic guitar, 1 keyboards, 1 trumpet, 1 Beat pad and an assortment of percussion instruments
- Members of the staff supported YP in their small groups

- YP from each group joined each other on regular intervals to exchange ideas to finally create one single musical piece (decided by YP)

Session 3 (4-4:30pm): Practicing their musical pieces to merge into one

- Few rounds of practices in small groups
- Participants come together in the music room and as a whole group put their musical creations into one
- Final performance was titled 'Safe space'
- Project members filmed final performance

Concluding session (4:30-5pm)

- YP invited to give their feedback. Different formats offered: written (on paper or online, prompted by questions), video recording filming each other
- Project team wrote on an A3 sheet an account of all the YP's had achieved during their one-day workshop.
- Concluding remarks from YP and staff members about the day.

Supplementary Material S4

<u>Analysis member-checking (17th January 2024, 6-8pm)</u>

Venue: Online over Zoom
Participants: 8 young people, aged 10-24, 3 facilitators
Plan: Familiarity to the analysis process, work on one term as an example

Introductions (5 mins):
Participants and facilitators do a quick round of introductions

Icebreaker session (10 mins):
- Young people split into two breakrooms of 4 people each with one facilitator
- Jamboard displaying 50 emojis
- Instruction to participants: "Categorise the emojis into groups and label each group. Create a minimum of 2 groups, no maximum limit. Use any criteria you see fit."

Discussing the activity, relating to analysis (30 mins)
Sharing thoughts on the activity, prompts included: What did you notice about the process? Did you invent rules? What rules did you use in order to create the different groups or sub-groups? Did you work individually? Did you check in with each other? Did you do it fully, collaboratively? How do you know, did you share your rules that you used in order to do these divisions.

Brief about thematic analysis (15 mins)
- Explained analysis process in a nutshell using examples from the transcript
- Three simple slides showing examples of:
  - What is a transcript
  - What it means to summarize text from the transcript (line-by-line coding)
  - How do we arrive at themes from codes/summaries developed from the transcript
- Discussing in relation to the icebreaker session

Main session
- Young people divided into 2 groups
- Facilitators ascribed to each group
- Discussions prompted by facilitators in each group
- Session recorded

First task on the term Safe space (20 mins):
Presented transcripts alongside its codes. Participants in each breakout room asked to respond on the Padlet with a thumb-up or down depending on whether they agree with the codes. Space provided to suggest alternative codes and/or add other comments.
Participants regroup and discuss together their observations.

Second task on the term Safe space (30 mins):
Padlet with themes, codes and quotes of the term Safe space shared with young people. Participants asked to match the codes with the transcripts. After that suggested to take some time to add comments and/or suggestions.
Closing session (10 mins)
- Whole group discussion
- Final thoughts
- Feedback

Analysis member-checking (1st May 2024, 6-8pm)

Venue: Online over Zoom
Participants: 5 young people, aged 10-24, 2 facilitators
Plan: Discussing analysis of further terms

Activities	Duration
**Introductions**<br>How has it been being part of this project? Use 3 emojis.<br>(2 mins to put in chat and 5 mins to share with others)<br>What is the plan for today? (3 mins)	10 mins
**Icebreaker**<br>Categorize the emojis (10 mins)<br>Observations and reflections (10 mins)	20 mins
**What is thematic analysis?**	5 mins
**Term 1: Resilience**<br>Comments and suggestions	35 mins
**BREAK**	5 mins
**Term 2: Mental health**<br>Match transcripts, codes and themes<br>*(Breakout rooms)*	35 mins
**Closing discussion**<br>**Observations/Reflections/Feedback**	10 mins

Icebreaker session (20 mins):

- Young people split into two breakrooms with one facilitator in each group
- Jamboard displaying 50 emojis
- Instruction to participants: "Categorise the emojis into groups and label each group. Create a minimum of 2 groups, no maximum limit. Use any criteria you see fit."
- Sharing thoughts on the activity using prompts (reminder of previous session)

Term :1 Resilience (35 mins):
Padlet with themes, codes and quotes of the term Resilience shared with young people. Participants asked to match the codes with the transcripts. After that suggested to take some time to review, discuss and comment and/or add suggestions.

Term 2: Mental health (35 mins)
Padlet with themes, codes and quotes from the terms. Young people were asked to match first the codes with the transcripts and then the themes with the codes. They were also encouraged to discuss, add comments and/or suggest alternative theme/s and code/s.

Supplementary material S5

Term tables
Descriptive themes and codes for each term separately for academics and young people

Term: Agency

**Academics/practitioners Descriptive themes**	**Sample of codes**	***Quotes***
awareness of choice and control	tied to conscious change	*if I've become conscious of the situation, I may not change it, but my consciousness about situation may may allow me to reframe how I deal in that situation and in that way that might be an agentic thing.*
	connected to choice and control	*Agency um is very much it. It is linked to consciousness and awareness and as soon as you have awareness then you have choice and that seems to me to be really important and that capacity to have choice and being also linked to control.*
	choice and control'	*I come from an arts background so I don't know how the term agency is being used in other disciplines or I may have misconstrued it because I'm bringing an art perspective to it, but from my experience agency is one of the most important words I associate with mental health or doing mental health research because it's about choice and control.*
different cultural value	cultural connotation/not always the most valued	*this is one term where I've seen real cultural difference. So for example in our work in India, when we try to put work on agency into school based interventions, they were like what you're talking about agency. Why would we want agency? Why are we not talking about relationships and doing things that are good for families and communities? And why do we always have to think about ourselves?*
	agency for what purpose - cultural difference	*More Eastern kind of countries, but there is much more of a focus on using them to like help with relationships. And, you know, rather than kind of um benefits for the individual. So it is quite different. Different approach, you know aims.*
sharing power	emancipation rather than empowerment	*I think when we talk about emancipation, we talk about, you know, all of us sharing sharing power, whereas with empowerment, it means I hold power. I give you few choices.*
	sharing the phenomenological field'	*But for me that sharing the phenomenological field, which is a really lovely way of putting it, that's where the agency is.*

attached to capacity	connected to resources and freedom to act	*Having the capacity, opportunity and freedom to act - from micro everyday actions to life choices.*
	dependent on the strength of the feeling	*I kind of almost see agency as a combination of feelings and thoughts actually so, and sometimes that can mean that you don't have agency because of the strength of your feelings, you don't feel you've got choices because you're too overwhelmed*
ties with mental health	associated mental health	*I come from an arts background so I don't know how the term agency is being used in other disciplines or I may have misconstrued it because I'm bringing an art perspective to it, but from my experience agency is one of the most important words I associate with mental health or doing mental health research because it's about choice and control.*
	connected to positive mental health	*And I think the minute that somebody feels they don't have a choice, they're not in control, then that's when mental health issues start to really, really rise up and so there's nothing more important or fundamental than individual agency or feeling that there are choices.*
**Young people Descriptive themes**	**Sample of codes**	***Quotes***
to do with control	control over change	*making change*
	control over own life	*Having control over your actions and the way you live your life*
not a common language	lacking knowledge of agency in research context	*I usually think about an agency just being another name used to refer to a company or an organisation*
	Agency left out of vocab in school	*you'd never really at school get told about agency so it's not something you would immediately think of.*
	unsure	*Action or intervention*
	alien concept (just like data)	*"I guess agency could just be a group by itself. Is like agency a community or people? I think data will go with agency."*

Term: Community

Academics/practitioners Descriptive themes	Sample of codes	*Quotes*
not always about connection	masking loneliness	*I was also thinking about the connection between loneliness and community, but also how community can hide or can be used again to mask issues of loneliness.*
	connected to loneliness	*you could easily feel very isolated, even though you've been identified as being part of a broader group. So I think something very interesting there*

		between the concept of loneliness and community and the definitions of community.
"kind of othering"	separating from others	*So the concept of a community was a way of kind of othering that, that group of people and precisely not thinking about them as individuals, but sort of like just thinking about them as a mass of people, that that were undifferentiated and the experience of being within that community was very, very different.*
	sense of a cult	*And then an interesting thing that through the process and the development teens into 20s and through the 20s, often a more and more not identifying with that community and considering it to be almost a cult.*
	category of box, not community	*I'm a member of. I'm a member of the black community and we all meet on a Thursday or 70 billion of us in the same space. No, we don't. But actually, when it's used in that parlance, it's demarcated on me because of my skin tone and phenotype. It's not a community. There's nothing about community in it. It's a category of box. So thinking about how, how that lands, how that operates and how our younger participants might view that.*
Bound by commonality	commonality	*a group with coherence of purpose OR identity OR characteristic.*
	"collective identity"	*Belonging to a group and have a sense of collective identity*
	feeling associated	*Something or someone you can relate to and feel a part of*
expectation of support	different support systems	*Feeling supported, part of different communities (family, family, place where you live, place you work*
**Young people Descriptive themes**	**Sample of codes**	***Quotes***
simply defined by closeness	unity as one	*a group of individuals which unite together as one*
	associated with connection	*multiple people who are connected together and have a sense of closeness. However, they are not necessarily alike. Could be a small group of friends or a big group of multiple people from various backgrounds.*
	associated with the closest	*...to community and I thought that... Well My G usually means like my guy or a friend or something and like when H is saying he sounds like he really cares about his friend and he is quite close to this person and it kinda reminds me of my relationships with like my parents and friends and things*

	people with close ties	*I'm quite close with my sister and things and I quite like, like the relationship and things like that and like My G is kind of like a term like used with like really close friends and things, so I just relate to it.*
	proximity with people/closeness	*"But connection and community are going to be squashed into one because they're quite similar. Sure. Why connection and community closer? Because community is the people around us, and connection you can connect with people around you. That's how I think about it in my head."*
commonality	common activity	*A group of people that come together as they have a similarity or commonality between each other. They come together and do activities together or share ideas for example*
	defined by purpose	*A group of people that come together for a similar purpose*
	common interaction	*A group who have something in common that they interact with each other based on their common interest/social interaction*
positive connection is not a given	positive or negative/connected through support	*People surrounding you that understand you and help you when needed even if u don't seek it out or verbalise it. You can also be apart of a negative community that deteriorates your mental health.*
	community can also have disconnects	*I'm like in the UK and then I've got India identity and how sometimes there's a disconnect in the community because it's like we're not one or the other. So like looking at the drums it's kind of like how it kind of almost doesn't match, that's what I'm trying to, figuring out how we can incorporate our India identity*
	community belonging could be a difficult manoeuvre	*we will also say our music tastes are very outdated because we only got into like English music 2000 we would say like 11ish and that is where our music tastes have kind of stuck because up until then when you're with your family and stuff you only listened to what your parents listened to or what was on at home and then once you started listening to your own music even then it was like what was on at that time and then it doesn't really move. So we were saying that it's really hard in ways to fit in on both of them, so yeah.*
	not a given, effort to fit in	*so do you know how you have your kind of classic movies that everyone watches like Godfather, Top Gun, all these that everyone's like "you must watch", we also have it in Indian culture and that's when you get to like 10/11 you've watched all of these certain ones, same happens in India. But then what we also have to do is kind of, it's then you've started at the start*

		where it's so far away that everyone's already watched these and you're playing catch up then, now you've got to get to that level on English movies and culture as well so it's like "oh I have no idea what I'm doing" kind of thing.
accommodating multiple identities	incorporating identities	*I'm like in the UK and then I've got India identity and how sometimes there's a disconnect in the community because it's like we're not one or the other. So like looking at the drums it's kind of like how it kind of almost doesn't match, that's what I'm trying to, figuring out how we can incorporate our India identity*
	multiple groups, some feels warmer than the other	*"Yeah. So we try and put like the Indian drums and bringing in like sound, which even though might sound a bit like off it's like meant to be there because it's like… They happened in the same space independently. "*
	community can mean bringing multiple identities	*"I think it really worked. Like you can tell that there's like kind of two, or like multiple layers there but like they go together, like it's not kind of jarring, it's like I think, is that what you were trying to get? Like you can tell but they're different… Yeah. …but it's like it's still okay here's a piece of music, it's like an identity, it's like all of these things contribute to make like one piece of music. I think, no, it sounds really good, yeah. "*
	odds can exist within the community	*And we also talked about sexuality because I'm bisexual and at home only my like parents know and if anyone else knew it would be "oh" kind of thing, and I think that's the other thing I would kind of resonate the drums with because like this is what everyone sees but that's what's actually how I feel kind of thing. But if it was to like develop I would definitely make the drums come a lot louder and then kind of fade back into even or like less so at one point.*
	intersections of identity	*We're saying how like it almost didn't match but I feel like we got this one idea where it kind of shows like, because all the theme was like a mix, saying like the relationship between like I'm like in the UK and then I've got India identity and how sometimes there's a disconnect in the community because it's like we're not one or the other. So like looking at the drums it's kind of like how it kind of almost doesn't match, that's what I'm trying to, figuring out how we can incorporate our India identity with [?? 05:25 background noise] and like, yeah, so that was [?? 05:29]*
space to connect	place for new connection	*Oh like, um, I feel I don't have a person to talk to, cry to, I just want to see what's new. New can*

		be about connection or community can't it because you're new to a new place of community connection?
positive space	a warm space	*It sounds like this music that would play like in the summer, you know, when you're just like going round watering your crops, like that's where it transported me, like just, yeah. No, I love it. It's really, it's, yeah…. Yeah. So peaceful... It's so Zen and just like warm and... like a safe space*
not all encompassing	bringing a part of you and not the whole	*when you're with, it's neither here or neither there when you're with the group, you're never yourself, you're always picking which side you're showing to who, like your friends, but when I'm with like my Indian friends it's full kind of thing and then when you're with your English friends it's then we don't talk about it, it's kind of you only see like one part of you and just trying to mix those two.*

Term: Connection

**Academics/practitioners Descriptive themes**	**Sample of codes**	***Quotes***
different context different meaning	technology vs real connection	*So the tendency for messaging not actually picking up the phone. We all know that from young people. But I'm I'm being told with dating apps that it's very rare to be able to actually connect with somebody on a date in real life, which is called IRL.*
	simply in network	*in kind of historical context that maybe maybe in a in a time when like young people are supposedly like, you know, a lot of them like just always connected or you know through social media whether whether being lonely, it is just problematised even more.*
	means to connection	*Feeling connected to a group, peer sub-group, connected to culture/broader communities through the arts and leisure pursuits*
common purpose/psychosocial	defined by commonality	*feeling like two people have something in common*
	something shared	*Shared understanding and shared goals*
	medium of connection	*Feeling connected to a group, peer sub-group, connected to culture/broader communities through the arts and leisure pursuits*
valuable asset/ to be preserved	hurtful when it breaks	*I chose this Jack Harlow song, Blame on Me and I related it to connection. He's talking about losing connection with his older brother. I found it interesting because I was... I found it quite hurtful to listen to but at the same time quite comforting and that. I found it quite difficult to listen to, it's hard to hear a relationship sort of like breakdown*

	Caring about something – to put effort	*I like the fact that he cared enough about it to actually talk about and for me, sort of like connection is just having like a rapport*
	lives linked	*connection is just having like a rapport, a relationship sort of like with somebody, you're kind of, your lives are sort of like connected and you're invested in each other and that's what connection means to me*
foundation of good mental health	associated with good mental health	*Connections are the forgotten foundations of good mental health*
**Young people Descriptive themes**	**Sample of codes**	***Quotes***
bound by trust	defined by trust	*Trusting another or multiple people with your feelings with full honesty*
	"example of a safe space"	*Allows for a sense of belonging and can be an example of a safe space.*
	'can be yourself'	*feeling like you can be yourself around others as there is a mutual understanding*
	respect and loyalty	*A sense of trust,respect understand or loyalty*
	bound by trust	*"About connection, um, like safe space can also be about connection. You can connect like… Yeah. Because like it's like when you have a safe space I think about you're with people that you trust."*
changing and developing	strong bond/connected with passion/ "bond over time"	*a spark or fire you feel between someone or something which can develop into a deeper passion or stronger bond over time*
	Always developing – old and new	*So I've picked Let Go by Central Cee and my word was 'connection'. I thought about connection because it's about looking back on memories and maybe thinking that you regret choices you made about connections in the past but I think the song really tells you to let go and kind of move on, live in the moment because you can't be regretful about the past when it's already gone, so just live in the future and obviously you have the chance to make new connections, so yeah.*
	rekindled ties	*So the song that I decided to go for was Metro Boomin called Hummingbird and I decide to link it to connection. The reason I thought it was good for connection is because it's used in a movie in which connection and in the scene the main character is connecting with somebody, he's meeting an old friend, so he's obviously forming connections with them and that's when the song is used to help with that, so yeah.*
	influenced by age and developmental phase	*if we reflect that so far as kind of like if you use the drums as the English side of my life and then the Indian when, I personally would make the drums get quite loud at one point because when I got to*

		around 13 to 16/17 I completely kind of stopped listening to Indian music, watching well not really movies but listening to Indian music even at home, I completely stopped speaking Punjabi majority at home and just went very much, these guys are gonna get what I say, coconut like [laughter], where you're very much, where you go to the extreme where you just disassociate with everything, but then since I left home and then came back I've kind of gone back into.
mutuality	reciprocating care	*Between people, it can be a feeling of closeness with someone. Caring for other people, looking out for them, wanting them to do the best that they can, and them feeling the same way about you, can show that you have a connection with them!*
	two-way understanding	*feeling like you can be yourself around others as there is a mutual understanding*
varied forms	healthy and bad/ connection not always positive	*So I chose a song Cherry Cola by Kuwada because... Oh, and I linked it to connection because I felt that it shows a way that proper connections should be and the difference between a healthy connection and a bad connection.*
	could be with an object	*he was talking about his bad connections of the past being like alcohol and how it's like confusing and things like that but and how his new relationship is like Cherry Cola and how it keeps him sober and refreshed*
bound by commonality	commonality	*Feeling similar to someone or something else. Having an affinity with it/them*
	common tastes	*I went from having all Indian friends to having all white friends and it completely like scared the hell out of me because I was like "oh my God, I have no idea what people are listening to" or movie, like movies, I've only just started watching English movies but when people talk about really simple ones like Top Gun and Godfather, I never watched them.*
	requiring commonality of experience	*"I'm lucky enough to have, you know, outside of school, yeah, I do dancing with these guys so I'm lucky enough to still have a community outside of school where I can like, you know, resonate and connect with, but inside school it's very much I feel like, I don't feel isolated but it's a similar situation where everyone has these kind of conversations that they talk about and I can't really like, you know, connect with that because I don't, I haven't had the same experiences, yeah, and it's a bit difficult when it's like you're the only one and you have no one else you can really like… Yeah. ...chat to. "*
feeling of closeness	comfort with someone	*Something you feel with people you are close to and feel comfortable with.*

	closeness in a community	*"But connection and community are going to be squashed into one because they're quite similar. Sure. Why connection and community closer? Because community is the people around us, and connection you can connect with people around you. That's how I think about it in my head."*

Term: Coping

**Academics/practitioners Descriptive theme**	**Sample of codes**	***Quotes***
masking	putting a lid on problems	*It it is it put putting lid on it. I might be coping because I'm not screaming now because I'm somehow managing not to scream. But does that mean I'm I'm OK?*
	camouflaging'	*Copying is not thriving- coping could be camouflaging*
self-regulation	strategies for self-regulation	*I put the bit about as strategists for self-regulation, so this puts a little bit more agency on it rather than sort of trying to protect yourself and bat something away.*
	a regular effort	*So with young people, these strategies, they definitely adapt to cope and a self-regulate in everyday life. So that seems to be distinct from resilience*
**Young people Descriptive themes**	**Sample of codes**	***Quotes***
regulated expression	suppression	*Dealing with negative emotions by suppressing them and refusing to outwardly share them to friends or family, even specialists that can be trained to fix such emotions.*
	expression of self	*How you express your self during a time of hardship*
	'keep going'	*Also with the coping you just keep going.*
an ability	controlled processing	*How a person processes and then deals with something they've been through/are going through. There can be both good and bad coping "mechanisms", I would say. For example, taking a step back and trying to process a situation through a different perspective, or taking a moment to breath, can be a better way to cope with something difficult than trying to ignore or be rude to people, or completely isolating yourself*
	connected to ability	*being able to deal with the situations life throws at you*
	'feeling able'	*Feeling able to deal with anything life throws in your way such as illness, life events, relationships etc.*
dependent on resources	connected to resources	*Having the resources to feel mentally and physically stable while dealing with challenges*

Term: Data

**Academics/practitioners Descriptive theme**	**Sample of codes**	***Quotes***
"can be anything"	different forms taken	*Actually it's interesting to see the different how how do we all define describe what data is and I think particularly makes me think you know how data can be anything in a way and working on a review with JH and AO and a colleague at* [city] *and actually you know it's using participatory video and then that whole review process it's really challenged my belief of what data is and what it actually looks like.*
	occurring and happening	*And and we will do we we will engage in some activities to elucidate that data and or it may be that data is just occurring or is being perceived as something is is happening. And I think an awareness of the opportunities and the tensions between those two states is something we need to be alive to.*
valued in terms of usage	meaning connected to purpose	*Um, if it can be applied, for instance, if it's scientific, if it's data that that's gonna be used for scientific research, and it's got to be able to be applied to test ideas and theories*
	"defined in terms of its usage"	*So it you know the the term data I guess again like the other terms it means it's defined in terms of its usage.*
	evidence connected	*So the more speculative the evidence in testing the rest of a claim, the less valuable that data is. I would say I would say.*
ability to triangulate	different forms used for triangulation	*But I think I think that's where I've probably found arts based stuff the most useful when it's used as a sort of part of a triangulation of arguments, if you like.*
	counter evidence (connected to triangulation)	*Sometimes they, they might just flip the narrative. Things that are taken for granted in science. Like I remember I was involved in a sleep project and and the key narrative is that everybody needs to understand sleep hygiene and autistic people might not understand sleep hygiene. Hence problems that are that one of the arguments anyway. And it came out through their photographs that they had very, very good sleep habits.*
**Young people Descriptive themes**	**Sample of codes**	***Quotes***
product of research	product of an investigation'	*The results or product of an investigation (usually) - something to take away and perhaps discuss again afterwards - or something that's been found out, something maybe new*

	by-product of research	*the information that is gained from research*
	by-product of evidence	*the end result/ what can be gathered from the evidence obtained*
valid information	connected to validity	*is often peer reviewed to gain multiple insights and make sure it is valid.*
	information	*Another name given to information. Used for studies.*
connected to stats	online statistics	*Whereas if someone said "I want you to grab the data", you know "okay, what I need to do is I need to go online and find statistics". You automatically like assume certain things go with it just because that's what you're like exposed to at school especially.*
	quantitative	*usually statistics or a type of chart to present evidence in a way that is easier to present and interpret*
confined to professional	Data as used by the other (researchers)	*But they're words that, I mean like other people use them but it's like especially when you're thinking about like a research project it's like there's the researchers and they're using the data*
	confined to academic work	*I think it's not a common word, especially because at our ages as far as research and kind of data is concerned your only use is in school*
	scientific	*And we had these three, data, evidence and research. I think they all go into a scientific category.*
	alien concept (just like agency)	*"I guess agency could just be a group by itself. Is like agency a community or people? I think data will go with agency."*
inefficiency of questionnaires	data should include reason	*I think like especially when you start something like CAMHS or therapy, you always start with the core ten questions and I feel when you do the questions over the phone you have a chance to kind of explain your reasoning in a bit, so if they're like, um, for whatever question, sometimes Rowley never kind of thing, you can kind of explain that, say "I'm saying never but, or I'm saying rarely, however it depends on this".*
	questionnaires as inaccurate means	*I have OCD and if I have a 1 to 10 question my answer is not only determined by the true answer, it's also well which number do I have to put, you know? If I'm actually feeling it's a 10 but I'm like well I actually need to put, you know, 9 because of my OCD, I guess like any research ever done on me has probably like got like a 10% inaccuracy rate because like I have to go to the nearest even number or like I have to make it balance [...] So like I don't know if I'm actually giving kind of accurate data because I'm always*

		like no, if it's a scale of 1 to 10 I can't use all those numbers. [Laughs]
to be analysed	information to be analysed	*the information gained from participants that is analysed quantitatively or qualitatively in order to understand what the data means in terms of the topic you are researching*

Term: Empowerment

**Academics/practitioners Descriptive themes**	**Sample of codes**	***Quotes***
genuine power	associated with genuine power	*I think for me the key thing is the notion of power within empowerment and understanding if you're genuinely trying to empower somebody that they might not end up doing what you necessarily want them to do.*
	not neutering power	*Co-option you know the the notion that you're you get everybody to do community arts because then you kind of like you bring everybody into the system and you're kind of like making everybody feel part of the community and if they feel part of the Community, they won't kind of come back and bite you because you know, so it's a way of kind of neutering power as opposed to empowering people.*
	authentic coproduction and co-delivery'	*Emancipation via authentic coproduction and codelivery- mentoring- wellness plans to allow people to reach max potential*
determined by external bodies	different meanings set by different external bodies	*to be really careful of because of how they are articulated by wider and different ecologies that sit around us like commissioning, like government policies, etcetera*
	demands a measure as proof	*It's a really tricky one because I get behind the ideology of empowerment and it sounds a lovely word. But in psychology it would be really, really difficult to get away with publishing any research where you claimed empowerment without having measured it.*
opportunity for involvement	motivator for involvement	*So a lot of the projects that I work on, there's a real kind of thin line between research and activism. And in terms of kind of activism and participatory arts, etcetera, the concept of empowerment and agency is essential because it's the real, it's a motivator. It's a way of getting people to be involved in it.*
	connected to leadership	*giving genuine responsibility and opportunities for leadership*
connected to agency	empowerment and agency - 2 sides of the same coin	*Yeah, that again, I think this probably links to agency, doesn't it? There's this is probably two sides of the same coin or to a degree.*
	percursor to agency	*offering choice to ensure agency*

not a given	not a holistic thing' - risk involved when it doesn't work	*It's not a holistic thing and it can be taken away in an instant where somebody tries out their power and it didn't work. And there's certainly massively disempowered.*
**Young people Descriptive themes**	**Sample of codes**	***Quotes***
drive towards positive change	empowering related to giving oneself and others	*Empowerment can be you pushing yourself to do the best you can - realising the good qualities you have to share and give to yourself and others can be extremely empowering and can help you to push yourself to new limits!*
	desire for change	*having a strong desire to make change and being driven by these internal feelings*
represented	feeling heard'	*Being and feeling heard. Feeling heard can be incredibly, incredibly empowering, and can be a huge boost for people who are feeling low and need a bit of a support or someone/something to lean on*
	represented and seen	*When u feel like Ur represented and this makes you feel seen and hence more powerful.*
control/conquer	control of own life	*Having control and power over your own life*
	negative? Advantage over someone	*When you feel a sense of pride due to having an advantage over someone else*
	ability to conquer	*feeling like you have the ability to do anything and can conquer whatever challenge comes your way*
	sense of power	*I picked It's About Drive because it empowers me because it talks about getting stronger, devouring and by devouring it means like becoming better, devouring things to become stronger, more powerful*
	in control of one's own life	*"I have a line. Go for it? Life is like a clear canvas. You just need to paint it."*
feeling strong	getting stronger'	*I picked It's About Drive because it empowers me because it talks about getting stronger, devouring and by devouring it means like becoming better, devouring things to become stronger, more powerful and yeah, that's why I chose devour because it makes me feel stronger, it empowers me, it makes me feel better, it makes me feel happier, so that's why I came up with devouring, so... No, that's why I came up with It's About Drive.*
	change through time to get stronger	*Like at the beginning of the video the woman was like sad and then she was really lonely but then she changed gradually through the rhythm, gradually through time and then she became more stronger*
positive self-evaluation	ability to overcome fears	*I've chosen it because like in everyday life you go through some kind of fight in life, like it might be with a friend, it might be with your family that you're going through something, which shows how you need to fight and overcome those fears.*

	contentment with self	*she was happy with herself and it shows that you shouldn't like judge yourself and you should be happy with yourself and you should be happy with who you are and yeah, that's it. It's about empowerment!*
	sense of achievement	*So isn't it like when you feel like you've done a good job isn't it? Empowered, you feel empowered.*
	self-confidence	*"What's that feeling? [No immediate response] It feels right but what is that? What sensation is that creating within you? Positivity in one way. Like when I hear that song I feel that I am something, like music helps me discover myself and find more things. So yeah."*
sense of security	knowing and being sure	*but feeling so true would, I feel like that would be about empowerment because you're empowered by what's true because you know that it's true.*
	safe and good	*"When you think of the word empowerment, what do you like, what do you feel? I feel like there's something that's good and that I feel sort of like safe kinda I guess. "*

Term: Evidence

**Academics/practitioners Descriptive themes**	**Sample of codes**	***Quotes***
associated with data	veracity of specific statement'	*Data that can provide information about the veracity of specific statement.*
	analysis of data leads to evidence	*analysis of data produces evidence of findings; the data provides the evidence and the research makes sense of it through an interpretative process*
	cross-comparison	*Analysis and cross-comparison of data sets to yield evidence*
	patterns in data/includes reflexive practice	*recognising patterns in data and interpret them- being reflexive about what interpretative model*
types of knowledge	different types of knowledge	*So I think when we look at evidence, we're looking at different types of knowledge here.*
	Nomothetic and idiographic	*So trying to find things that hold true across populations and then looking at things that might be more distinct but nevertheless important.*
	validity and reliability of different disciplines	*also the words valid and reliable on how they're interpreted differently in different disciplines and subdisciplines.*
meaning determined by external bodies	external bodies define	*It's probably stating the obvious, but I think what counts as evidence is really defined by not only our subdisciplines, but by journals and funders and what they feel is valuable*
	fluid based on what is told	*So um, I think I'm struggling to say what evidence is and isn't because I almost feel like*

		we're told what it is and isn't by those different areas.
**Young people Descriptive themes**	**Sample of codes**	***Quotes***
backs up claims	connected to validity	*A scientific or valid piece of information to back up a claim or finding.*
	proof	*It's usually considered something that's used to prove something else*
	assuring discovery	*Something that may provide confidence and assurance for a discovery or just a regular claim.*
	factual statement	*facts to backup a claim or statement to ensure it is true to others rather than false*
professional	scientific	*And we had these three, data, evidence and research. I think they all go into a scientific category.*

Term: Loneliness

**Academics/practitioners Descriptive themes**	**Sample of codes**	***Quotes***
Not fitting in	Not seen/understood	*I think for me the idea of visibility being seen being understood is the experience of not being lonely.*
	seen as different	*That's I find that helpful to think about that when you do feel lonely, you feel that nobody gets you, that that you're very different.*
	outsider	*feeling disconnected and having a sense of being on the outside*
Part of life course	outcome of a developmental phase	*And it's partly we understand it in psychology as partly an outcome of that developmental phase of finding your place in society, but I'm not sure that that's a good enough explanation actually for for what young people feel.*
	inherent aspect	*philosophical position that we all have to get more comfortable with loneliness. It's not something we have to eradicate. Um and maybe it's how we explain what, what loneliness is, because we're we're all inherently lonely as human beings cause we can only fundamentally know ourselves.*
Disconnection	not feeling supposed connection	*You can feel even more lonely if you're like, supposed to be like connected to this community. But you know, you don't feel you don't feel that.*
	isolation	*feeling isolated from peers*
	lack of connection	*Not feeling a sense of connection/involvement in society or with peers*
problematised	having 'lethal consequences'	*loneliness has lethal consequences- many types of loneliness- cultural, peer, family etc*

	social media influence of meaning	*historical context that maybe maybe in a in a time when like young people are supposedly like, you know, a lot of them like just always connected or you know through social media whether whether being lonely, it is just problematised even more.*
**Young people Descriptive themes**	**Sample of codes**	***Quotes***
Lack of belongingness	lack of belongingness	*feeling like you don't belong somewhere - feeling left out from a community or a group of people*
	feeling of an outsider	*A good phrase to think about would maybe be 'feeling alone in a crowded room' - being surrounded by people but maybe not feeling included, not feeling surrounded by people who you feel care about you, not feeling like you "fit in"*
Lack of companionship	feeling of isolation, not just alone	*usually associated with feelings of isolation but being alone doesn't necessarily cause loneliness*
	"no one to speak to"	*Feeling as though there is no one to speak to or tell your problems*
	emptiness	*When u feel empty even if u try ur best to change this. It can be difficult to express why u are feeling lonely or to even understand how to stop feeling lonely.*
	Lack of companionship	*Lack of companionship*
		The feeling of always being by your self and having no one to rely on
	Only one's own company	*the song that I chose was Lithium by Nirvana and I chose this song because to me it says quite a lot about loneliness. So in the lyrics the singer talks about how, you know, the only friends he has are the voices in his head and how he's effectively alone*
	no one to be open to	*So we've gone for loneliness. I don't feel that I have a person to have a good cry to. I just want to see what's new. It's a safe space. Ya, that's true.*
a feeling difficult to communicate	no one really understands'	*So in the lyrics the singer talks about how, you know, the only friends he has are the voices in his head and how he's effectively alone and no-one really understands what he's going through and from the singer's point of view it kind of describes his struggles with mental health*
	unable to understand the feeling	*In a safe space, yeah, that's true, um, but why do I always feel so blue, going back to loneliness*
common experience	common experience/ not alone in loneliness	*and I've always felt a deep connection to The Smiths and their lyrics, especially through Morrissey's openness to how he was feeling at the time of writing and throughout his life. The song specifically links to the term 'loneliness' because it highlights that while you can feel lonely you're not alone in that feeling,*

		so it reminds me that I'm not the only person that feels lonely
	sense of community	*And depressed and sad and all those other feelings I get along with that, so it almost gives a sense of community within that, a community of loneliness, especially talking about suicide, it's a very personal topic and for someone to speak about that makes you feel less lonely about those things, so yeah, that's how that song fits into the term 'loneliness'.*
	commonality is empowering	*I think the actual kind of song itself draws on lots of different topics about like fighting helplessness and it reminds me that like I'm human and everybody's a human and everyone's alone, so it's quite empowering.*
low feeling	depression with loneliness	*And depressed and sad and all those other feelings I get along with that, so it almost gives a sense of community within that, a community of loneliness*
	tragic and painful'	*it definitely brings out a lot of emotion and explores the more like tragic and painful sides to loneliness, especially someone who's experienced and been through a lot of loneliness for a long period of time.*
	negative feeling	*sad and ominous*
lot of emotions attached	lot of emotions tied to it	*chose Mortal Man by Jeremy Loops because it related to loneliness because the first time that I listened to it was kind of, I think it was near Covid but either way I'd been feeling very lonely and it just came up after a song, it automatically played and the first time I heard it even and every time I hear it even now, it like instils quite a lot of emotion in me*
	chaotic feeling	*I chose Awakening by Ichika Nito and I linked it to loneliness and I felt, I felt like it linked to loneliness because of the contrasting harmonies in the song. The whole song seems and feels quite chaotic*
	a lot of emotion'	*it definitely brings out a lot of emotion and explores the more like tragic and painful sides to loneliness, especially someone who's experienced and been through a lot of loneliness for a long period of time.*
accepting as part of life	accepting and enjoying (confused with being alone?)	*how he's effectively alone and no-one really understands what he's going through and from the singer's point of view it kind of describes his struggles with mental health and personally, it resonates with me because I personally am trying to get more comfortable with being by myself because sometimes I do enjoy my own company*
	necessity of lone time	*I was taught that I had to enjoy what other people enjoyed and that I had to enjoy some time with other people, so now that I'm older I'm trying to give myself the space to be by myself if I need to.*

Term: Mental health

Academics/practitioners Descriptive themes	Sample of codes	*Quotes*
continuum and ever-changing	state that continuously changes	*I agree that mental health seems synonymous with mental illness. However, we can view mental health as a scale experiencing periods of both positive and negative mental health. It is not a static state and is individual.*
	ever present state	*diagnosed or defined like depression, say sometimes what what happens is people say I've got mental health whereas actually they've got mental health all the time.*
not visible in the body	connected to experience and emotions	*Processual experience (conscious and non-conscious) - a particular primary affective background (mood) plus shifting, positive and negatively valenced emotions*
	cannot be derived from physical aspects	*Again, if we went back to definitions and and labelling nomenclature mental health is the health of of the of the cannot be derived from looking at the body, is it not?*
disorder-focused	clinical connotation	*health definitely obviously has a very strong clinical connotation*
	associated with mental disorder	*Yeah, of course there are risks in um individualising conditions, but there are also risks in abandoning the language of mental health, and in that you may trivialise the real suffering and distress that mental disorders do create.*
	'clinical practise'	*I wonder if the wellbeing side of kind of community arts is much more about kind of people keeping fit and being healthy and creating community and all that kind of stuff. Whereas when people talk about mental health and arts is that much more kind of music therapy, art therapy, that kind of, you know, specifically kind of a clinical practise in terms of the art.*
	originated from psychiatry	*mental health has emerged from psychiatry, whereas wellbeing has probably more emerged from positive psychology and the idea is that we should be looking at not just what predicts disease, but also what predicts flourishing and doing well and having a good life.*
	'a condition'	*maybe that I have a condition, a lifelong condition, that doesn't have a cure. Um, and that might be synonymous with my mental health. I might have, I don't know, borderline personality disorder or something like that. I don't go into the debate about whether that's a thing. I may have a situation, and if I'm managing it and doing well in the world, then*

		you could say my wellbeing is is good. But my mental health in that context is extant.
dichotomy	opposite of healthy	*the absence of mental illness*
**Young people Descriptive themes**	**Sample of codes**	***Quotes***
maintaining wellbeing/ wellness	'how well'	*The study behind how well you are mentally and how the things around you make you feel*
	connected to wellbeing	*Emotional ,psychological and social well-being*
	how well one is dealing	*How someone's mental state is and can be affected by experiences they go through, as well as how well a person can/is able to deal with certain things they go through.*
	connected to 'how well'	*How well u are doing mentally. Mental health can change rapidly and is not fully understood by most.*
	how well you're coping, not feeling	*Yeah. So here we've got evidence, data, research and agency, which we feel are more scientific words. Here we've got mental health, coping, wellbeing and safe space, which is it's all to do with mental health and how you cope with things, how you're feeling. Oh no, how to cope with things. Loneliness, connection, community, empowerment and resilience, they are all how you feel, you can, they're feelings you can feel them. And then stigma is ones that we're not sure what they mean.*
a state of mind	present and always changing	*How well u are doing mentally. Mental health can change rapidly and is not fully understood by most.*
	constant state	*everyone has mental health- some people's might be 'better' or 'worse' than others'*
	health of your mental state - biological or otherwise	*How healthy your mental state is. This can be more subjective in terms of how someone feels but also biological in terms of diagnosing someone with a mental health condition.*
	mental state	*How someone's mental state is and can be affected by experiences they go through, as well as how well a person can/is able to deal with certain things they go through.*
	changing state	*The kind of fade-in fade-out, so maybe that, if they could, because mental health fades often, like switches up, so it can fade-in or fade-out.*
emotion related	not visible	*a reflection of your inner being through things like emotions that are not necessarily visible from an outside perspective*
	emotional state impacting personality and behaviour	*Fluctuations in emotions that can either positively or negatively effect thoughts. Side effects, especially on the negative side, can have detrimental affects on personality and behaviour.*

	fluctuating emotions	*Fluctuations in emotions that can either positively or negatively effect thoughts. Side effects, especially on the negative side, can have detrimental affects on personality and behaviour.*

Term: Research

Academics/practitioners Descriptive themes	Sample of codes	***Quotes***
structured approach	'air of formality'/Paradigm/context driven	*And it has a play. It has a an air, which the term research has an air of formality about. It sits in certain contexts and paradigms.*
	structured/ curiosity	*Investigate a topic or problem through a process of structured enquiry using a range of sources. Being curious!*
	systematic way of testing and producing data	*Systematic activity to answer a specific empirical question using logic and data.*
different from intervention	Differentiating between research and intervention	*I mean, you know the actual process of putting the bid together was really a working example of why we needed this project because there was a real sense of a difference, particularly between the concept of research and the concept of an intervention.*
	Research vs Evaluation of intervention	*But understanding that the research is the kind of like, the more abstracted insights that emerge from an intervention emerge from the evaluation of an intervention as opposed to the evaluation of an intervention itself.*
testing/innovating	"Aimed towards a new treatment Connected to innovation"	*I think quite often I'd see the term as and something looking at developing a new treatment or developing a new sort of way of working with patients.*
	Connected to the process	*Testing ideas/theories, interpretations in the context of some kind of external reality. Investigating, observing, discovering*
influence on research concept/practice of external bodies	Connected to what REF outlines as research/ oppressed by expectations	*That's probably because it has a certain set of expectations we associate with the research excellence framework, you know, so it's it's sometimes feels quite oppressive.*
	Rules set to guide production of data	*But always on there that we have to work within a set of constraints in order to make the research matter in terms of producing data.*

understanding something	Research=understanding	*Discovering facts on this kind of a sense of external reality, and I guess I have a slight problem with that and I think the word understanding is probably one that I'm more comfortable with that research and help us to understand*
	Connected to outcome	*Looking to explore or find out more about something we don't yet know. Being open to new ways of thinking or working*
dependent on the field	Different meanings to different fields/rigid and robust/connected to intervention/contestable by different fields	*And it's really interesting just going to the OED and looking at it, it seems formal. It has [...] an influence of rigidity and robustness. And both of those things, I think, speak to some of the other unspoken around some of the terminology. So research sounds organised and has a status, whereas rehearsal might not or play might not so.It was really interesting that in in talking about this now, we've we've already said the word intervention and it seems like in health in maybe translational science and in [...] other arenas, even that word is is contestable contested and means different things depending on how it's applied.*
	Connected to data and what it means to different people	*I remember I was once in a project I was in the middle of collecting photo voice data and the the key person above me was excited was like, this is beautiful, beautiful stuff. It helps me a lot to understand. Now, can you run a survey so we can have some proper data? So what is proper data? You know, that's how serious alternative. If you like it. Data are are are taken that that's also something to to consider.*
	broad and amorphous'	*That was a little about what it ought to be, what it ought not to be, but what it is is an incredibly broad and amorphous thing that loads of people doing different ways and trying to derive the core.*
phenomenology focused	connected to lived experience	*things that we would research are so complex and so shifting and culturally bound and time bound, it's there is no fact with an understanding of well from my perspective from phenomenological perspective in that*

		how people live in the world and how people experience the world.
	connected to phenomenology	*Being curious to find out about a phenomeno from people who experience this phenomeno and see what scientists and therapists may learn from that. Passion to see change/real world impact.*
	phenomenological	*so as a researcher everything I've done is phenomenological and I said as a researcher there, and when I hear the piece I'm hearing something which is a phenomenological experience of research*
	phenomenological and yet feeling 'researched on'	*so as a researcher everything I've done is phenomenological and I said as a researcher there, and when I hear the piece I'm hearing something which is a phenomenological experience of research, is what it feels like to be researched on. So that's fascinating. Very powerful*
**Young people Descriptive themes**	**Sample of codes**	***Quotes***
searching and concluding	connected to conclusions	*Searching a conclusive answer through findings, can be on the internet or from media such as books*
	searching to support a goal	*searching and finding anything necessary to aid yourself*
finding and developing ideas	connected to outcome/discovery	*a way of discovering new things and ideas.*
	knowledge generation	*conducting investigations into a specific area of interest to try and improve and add onto ideas already developed or develop your own ideas/concepts from the research. Overall finding out more about a certain topic*
	investigating variables	*inquiring into a topic and considering a range of factors/ variables that might influence the thing being researched*
professional term	not commonly used/ specific purpose	*But they're words that, I mean like other people use them but it's like especially when you're thinking about like a research project it's like there's the researchers and they're using the data*
	confined to academic work	*I think it's not a common word, especially because at our ages as far*

		as research and kind of data is concerned your only use is in school
	excluded/ not inclusive/ separate from it	*So yeah, I guess it's about how people just don't necessarily understand and they can feel a bit kind of excluded from the researchers because it's not necessarily, even if it's like is inclusive they may not necessarily think of themselves as kind of being part of it.*
	Demarcation between researcher and participant	*"Yeah. It still feels like a them and us. Yeah. It's like they're researching you rather than you being like a participant."*
	scientific	*And we had these three, data, evidence and research. I think they all go into a scientific category.*
related to stats	more statistics than qualitative work	*so now I think of it a lot more, more as well in quantitative terms because we do that a lot more than qualitative, so it's more like numbers, statistics*
	associated closely with quantitative method	*more as well in quantitative terms because we do that a lot more than qualitative, so it's more like numbers, statistics, you know, recruiting participants, giving them questionnaires, things like that.*
	doubting arts-based methods as research	*So when like we're talking about like this was research, I was like "well this is not the research that I know of" but, you know, I was confused and like how is this qualifiable? How are you measuring whatever we're doing if you know what I mean?*
	connected with numbers, measurable	*So when like we're talking about like this was research, I was like "well this is not the research that I know of" but, you know, I was confused and like how is this qualifiable? How are you measuring whatever we're doing if you know what I mean?*
	Research= 'measurable'	*And that's the other thing, everyone takes research as measurable, even in something as simple as adverts, 76% of a hundred and eight people said this toothpaste was good*
	in percentages	*And that's the other thing, everyone takes research as measurable, even in something as simple as adverts, 76% of a hundred and eight people said this toothpaste was good, it's always*

		percentages rather than saying that "oh some people said it worked but the people who said it didn't work said it because they didn't like the taste"
more than just numbers	Meaning changed by knowing more through degree	*Because being exposed to like a lot of psychological research through my degree it's sort of changed the meaning for me, so now I think of it a lot more*
	Starting to see reason as research rather than just a number	*it's always percentages rather than saying that "oh some people said it worked but the people who said it didn't work said it because they didn't like the taste", whereas this is more the reason why behind it rather than just a number.*
what you do on the internet	finding on the internet	*when you get told to do an essay you think loads of paper, writing, when you get to do research you think going looking on the internet, finding certain thing*
	looking on the internet'	*when you get told to do an essay you think loads of paper, writing, when you get to do research you think going looking on the internet, finding certain thing*
research as questionnaires	research as requiring survey	*I mean I know that sometimes you have to have like a tick box exercise, like obviously you have to have surveys and stuff, but sometimes it does, you know, it feels like you're, especially when it's something neuro mental health related it feels like you as an individual is being, how would you possibly reflect an individual and all of their experiences and everything that's going on in their head in like, you know, a list of statements on a piece of paper*
	dissatisfied with use of survey/ not representative	*I mean I know that sometimes you have to have like a tick box exercise, like obviously you have to have surveys and stuff, but sometimes it does, you know, it feels like you're, especially when it's something neuro mental health related it feels like you as an individual is being, how would you possibly reflect an individual and all of their experiences and everything that's going on in their*

		head in like, you know, a list of statements on a piece of paper
	when measured in numbers - not representative	*I know like myself because I've had to fill out stuff before like in regards to my mental health and like therapy and stuff and it is really difficult like to sit down and judge on a scale of 1 to 5 to what degree this, you know, how often do you feel this way? It's really difficult and I think sometimes I, you know, you feel like you're not being represented because you're trying to get this information down and, you know, you want to speak out and you want to share your experiences and your story but you can't do that on a piece of paper.*
	research needs to incorporate reason	*So in some ways like I get why we need to do research and have like questionnaires that are on paper but I feel like even if it was just a box at the end maybe say "if you wish you can justify your answers", even something very simple like that because it just means I can explain why I've put that*
feared entity	bold and dark'	*I think I'd have a very strong bass, like "dun". I know you have the clicking clock, ticking clock but I'd have like something quite like dull or firm, even if it was just like a drum just going "dun, dun, dun" in the background, just so it gives like a, I'm going to say hardcore but you know like really bold like dark kind of thing.*
	anxiety from chaos	*I know it takes a while to like come up with all these and stuff but like maybe to like, if like any, like the anxiety of any kind of like situation, maybe to, like kind of like emotional feelings, you know, maybe add like contrasting like melodies or harmonies and things like that to give it like kind of like a chaotic maybe kind of feel, something like that.*
	research is frantic'	*I really like the way you structured it as well because you were going with research and research is frantic anyway.*
	erratic yet structured	*You could have really easily just had loads of sounds, like erratic sounds just piled on top of each other on loop*

		but you went with structure, which was really nice. But you could have it, if you were like to develop it have at one point where everything just goes erratic, like one on top of the other really fast and then kind of slows back into what you started with.

Term: Resilience

Academics/practitioners Descriptive themes	Sample of codes	*Quotes*
holding people responsible	negative connotation- not needing help	*lots of my work resilience can be used as a synonym for ripping people off because you can be around about trying to say, well, it's really good, people are resilient. Therefore, we don't need to worry about them.*
	asset or a problem?	*Resilience is a way of teaching people to put up with situations that they shouldn't put up with. And I wonder in terms of arts based in terms of mental health research, how much is that... you know how much is a focus on resilience, actually a problem as opposed to something that we should be instilling in people.*
	a quality in some people	*But I guess the the new exposure that I've had which is making me slightly more pensive may come from a more scientific and all clinical and Orpheus therapeutic environment where there is a curiosity about why a a series of events have taken place that are challenging and why some individuals exposed to that situation are not destroyed by it.*
	negative connotation - a characteristic one ought to have/associating with personal strength	*in terms of resilience um like said kind of putting it on the person that it's kind of you. There's something wrong in a sense that you're not resilient enough even though the situation itself, you know, could be.*
	term politicised	*I hate the term and it's but also it keeps coming back around and I think what strikes me is how politicised it has been because I think it was the Cameron government who were talking about wanting to develop grit in young people?*
no room for hope	made to accept and cope	*And all their masking that they're coping and coping is so far from thriving and It's it's, it's just almost like asking, particularly with with some particular mental health interventions that are very cognitive or very behavioural asking people almost to, to accept their oppression, to accept the issue and and and get on with that.*

	stripped from hope	*strips people up completely from hope and self-esteem*
	hope instead of resilience	*Hope may be a better alternative than resilience- resilience gives no hope for change of circumstances and places emphasis on person tolerating*
a way of thriving	being able to safeguard	*Yeah, no, I just, I just think a part of sort of being resilient is like being able to kind of safeguard yourself in terms of that you can like, resist or challenge if situate if if you're if demands are unreasonable or situations unreasonable.*
	capacity to thrive'	*Resilience for me is the link because it spoke about how, irrespective of what's going on and what was going on in my life, I was having capacity to thrive and come through it*
	drive towards self-care	*I discovered the tune at a point in my life when I was beginning to find my identity, my tribe, my people, my community and then at the point where the track drops was me just letting go and coping and taking care of myself.*
	managing to cope	*Participants, usually in research about, you know, do they feel resilient? They rarely use that word. They say, well, I'm just about managing or I've just coped, or I've got through something.*
	what doesn't kill you, makes you stronger'	*in the strictest sense in psychology, resilience emerges from a diversity. It's a kind of what doesn't kill you, makes you stronger. That was the original interest in it*
	adapt as well as 'push back'	*It's not just a case of putting up putting up with it and learning how to adapt always, but being able to sort of push back when appropriate.*
**Young people Descriptive themes**	**Sample of codes**	***Quotes***
retaliate	bounce back'	*being able to 'bounce back' from a more negative situation*
	not give up, fighting	*When u keep fighting back and dont give up.*
ability to cope	maintaining wellbeing despite adversity	*Being able to cope with negative or challenging emotions and still be able to do your task effectively*
	reaction to situations - associated with ability	*the aftermath of your reactions to emotions and situations - similar to coping but resilience is the fact that you are able to overcome these problems*
	'being able'	*being able to 'bounce back' from a more negative situation*
	A treasured trait – rewarding at the end	*There are going to be moments in life where you look back and you see that being resilient was the best thing*

moving on	adapting and changing'	*Adapting and changing to challenging changes and experience*
	dealing with a painful memory	*I chose Helmet by Steve Lacy for resilience because I think that the song is about him reflecting on a past experience and the memories that were good in the moment but now he's seeing them as painful and something he has to let go of, so I think that it represents resilience as he's becoming stronger and overcoming these memories and letting go of someone that he used to love so dearly.*
	let go	*so I think that it represents resilience as he's becoming stronger and overcoming these memories and letting go of someone that he used to love so dearly*
	Getting on with life despite hopelessness	*I've chosen the song Happier by Marshmello. I've connected this song to the word 'resilience' because I feel like the word resilience to me means to continue going on in life because there are going to be moments in life where you feel hopeless*
associated with strength	becoming stronger'	*so I think that it represents resilience as he's becoming stronger and overcoming these memories*
	Contentment with one's own strength	*I've connected this song to the word 'resilience' because I feel like the word resilience to me means to continue going on in life because there are going to be moments in life where you feel hopeless, that you feel like nothing's going to get better but at the end of the time, at the end of the whatever in life, whatever moment in life, you're going to look back and you're going to feel happy that you've gone through it.*

Term: Safe space

**Academics/practitioners Descriptive themes**	**Sample of codes**	***Quotes***
creating boundaries	antithetical to production of good art'	*this is my hobby horse really in terms of arts based practises where safe, there's an awful lot to talk about these projects as providing safe spaces, which in lots of ways is antithetical to the production of good art, if you like, because quite often art is about rupturing safe spaces is about taking risks about pushing boundaries.*
	external bodies determining meaning	*the last two weeks in different projects, we seem to have been really wrestling with ethical bodies that are determining what is safe and what isn't safe, and that's proving to be very, very constraining.*

	someone to be liable for safety	*I'm I'm not not saying we should be bound by them but there is some sense of liability or in a tribunal situation who is gonna be sitting there and I you know I know we shouldn't be dictated by these other authorities ahead you know in ethics committees but I think we do need to bear that in mind that actually somebody, somewhere, it is gonna be liable should something happen.*
associated with trust	sufficient trust'	*so you've built sufficient trust and kind of, you know, feeling by the young people that they're kind of held enough to to actually then be able to challenge them, to bring about more positive developments. So it really is kind of key to, you know, that they you are in a position to push more.*
	trust and care	*two stage process that you initially… um, you build kind of trust, and yes, some people sort of describe that synonymous with safe space, but they don't mean it safe in the sense that nothing happens. It's safe, more in the sense that you first create that trust and that care.*
	enough trust to motivate change	*enough understanding between the people involved that they then are able to kind of engage in though this kind of challenging processes of change.*
	private space in public context	*Trusting a particular environment - customising experience - creating private spaces in public contexts*
no universal connotation	differing perspectives on what is safe	*at the heart of it, it's defining what a safe space looks like. And I think that looks different to different people. It looked different for different adults. It looks. And it will look different for different young people*
	fit to the needs of the stakeholders/no universal meaning	*So and like you said with professionals might see that safety as safety planning and and things like that, whereas we've spoken to a lot of participants who actually say they want to be able to talk about their self-harm.*
comfort	void of distressing experiences	*A space either physically or emotionally where you feel most comfortable and the least at risk from distressing experiences*
	accommodating	*a space where your abilities are recognised and your struggles are accomodated*
	space away from fear	*A place where you go when your freightened*
better terms to use	optimal allowance of activity rather than 'safe space'	*it's interesting looking at how these term, how those terminology is being thrown around and I think it would be productive for us to identify environments where are God this might sound… but environments that allow for an optical, an optimal allowance of activity and just get away from any safe unsafe... because I think we could*

		talk about…some of the ingredients that drop into that space to to mean that we've taken care of like risk assessments and safeguarding etcetera
	compared to liminal space	*there seemed to be a sort of either a juxtaposition or a sense that they were synonymous comparing the notion of a safe space with a liminal space […] for me, that's they are very, very different things you know, and in some ways I'm more comfortable with the notion of a liminal space, because it's it sounds like it's it's it's an unstable space of transmission*
	care rather than safety	*I would be happier if we outlined our situation that showed that we were… focused around aligning things to put to, to take care of everyone embarking in the activity rather than having to waste time and talk about safety.*
**Young people Descriptive themes**	**Sample of codes**	***Quotes***
comforting	space away from stress	*somewhere you can get away from any stress or issues you have in your life*
	reflect without risk	*where you feel comfortable to reflect on what has happened or take your mind off of it.*
	comfort with people around	*A space that comforts an individual. It doesn't always have to be a physical location, it can be surrounded by certain people or it could be changes in temperature or humidity or noise level*
	a happy warm space	*It sounds like this music that would play like in the summer, you know, when you're just like going round watering your crops, like that's where it transported me, like just, yeah. No, I love it. It's really, it's, yeah.*
trusting environment	associated with trust	*Where you feel safe to talk about things and somewhere with people you know you can trust*
	confidential	*Non judgemental zone that is confidential*
	feeling of being 'protected, calm and loved'	*somewhere you feel protected, calm and loved. I don't think it necessarily has to be a physical location but maybe just being with a certain person can be a safe space. The sense of knowing everything is okay when you are in that space*
	associated with trusted connection	*"About connection, um, like safe space can also be about connection. You can connect like… Yeah. Because like it's like when you have a safe space I think about you're with people that you trust."*
not be judged'	space to be yourself	*A safe space can be a place where someone feels comfortable and calm to be themselves and be open. You can maybe also have a safe space with someone, where you feel open and free to be*

		yourself and let out anything that's been bothering you. Or maybe even just a space where you feel happy
	void of judgement	*A safe space is somewhere where you feel comfortable speaking about your feelings and where you are confident that you will not be judged and you are free to be yourself.*
relatable environment	relatable culture/communtiy	*when you go into like your safe space, majority of us tend to fall back to listening to Indian music rather than English music and that, and safe space at home especially is trying in your room to be alone but in general that when you put music on you go back to rather than English music.*
	what you are used to	*when you go into like your safe space, majority of us tend to fall back to listening to Indian music rather than English music and that, and safe space at home especially is trying in your room to be alone but in general that when you put music on you go back to rather than English music. Because for majority of us English music really wasn't a thing that we listened to until you got to like 7 or 8 because you used to listen to what your parents listened to, so from*
freedom	'do what you want'	*"We're all open here so… Yeah. ...you can do what you want. We're all open here so… Yeah…you can do what you want… Aw. Okay. It's a safe space."*
	freedom of action	*So we've gone for loneliness. I don't feel that I have a person to have a good cry to. I just want to see what's new. It's a safe space. Ya, that's true.*
connected to positive mental health	basis for good mental health	*Uh, safe space and mental health. I think they go together because you need to feel safe with who you have around you and can help with your mental health feeling safe.*

Term: Stigma

**Academics/practitioners Descriptive themes**	**Sample of codes**	**Quotes**
picking on traits	demarcation	stigma troubles in two directions. One is it is it uh the the process of demarcating someone or something? And the other direction being the the management. uh exposure and or implications of that demarcation
	purpose beyond control	Being treat in a certain way for a factor that is out of your control
	picking characteristic	Being viewed only in terms of a stigmatised characteristic rather than as a whole person

intolerance to divergence	reaction to divergence	negative sociatal reactions to an individual expressing divergent actions/thoughts
	defined by traits and shaming/ "power practice"	Intersectional stigma is a common experience for myself and my colleagues and is concerned with gender, age and identifying with a marginalised group. It is inextricably linked to shaming, power practices
**Young people Descriptive themes**	**Sample of codes**	**Quotes**
negative stereotype	stereotyping	I usually consider stigma to be negative feelings or stereotypes attached to ideas or beliefs. Someone might also have stigma around certain things because of personal bad experiences that they've been through
	taboo	a negative stereotype or taboo surrounding something- there is often a stigma around mental health, especially with the older generations
creating fear	associated with "dislike and fear"	negative connotations and prejudice that create an air of uncertainty, dislike and fear around a topic
unfamiliar concept	unknown	"Yeah. Then stigma and that's all we have left. I think it goes with scientific. […] Like I don't know what stigma and agency is really."

Term: Wellbeing

**Academics/practitioners Descriptive themes**	**Sample of codes**	***Quotes***
"nebulous concept"	broad and abstract	*I think well-being for me is a more nebulous concept is quite broad term*
	different meanings unlike mental health	*I think it means different things to different people in a way which perhaps mental health doesn't. Or maybe it does to the same degree, I don't know.*
	encompassing all aspects of mental health	*Overall mental health, holistically, taking account of social, familia; cultural contexts that contribute to wellbeing*
something you can control and do something about	wellbeing is actionable	*And with mental health, I know again, having worked with a lot of young people, mental health seems to be more inside your head and something that just is there and it's kind of not your fault type thing. And wellbeing is often much more connected. And to do with actions and activities and much more situated in the world.*
	'people keeping fit' in community arts	*So I wonder whether you know… different community and participatory arts practises are used differently depending on whether you're about mental health or wellbeing, so I wonder if the wellbeing side of kind of community arts is much more about kind of people keeping fit and being healthy and creating community and all that kind of stuff. Whereas when people talk about mental*

		health and arts is that much more kind of music therapy, art therapy, that kind of, you know, specifically kind of a clinical practise in terms of the art.
	regulated wellness	*it might be anything from keeping fit to to painting and using music.*
	effective functioning/ some control	*Feeling good and functioning effectively, informed by degree of control (autonomy), resilience, feeling valued, self-valuing and getting absorbed by things/openness to experience*
	ability to maintain wellness	*Keeping well in the face of life events that we all face daily!*
'having a good life'	good mental health leads to wellbeing	*that when we talk to them about mental health, they might think of anxiety for example and not the you know, good side of keeping up with wellbeing.*
	maintaining positive mental health despite exisiting conditions	*maybe that I have a condition, a lifelong condition, that doesn't have a cure. Um, and that might be synonymous with my mental health. I might have, I don't know, borderline personality disorder or something like that. I don't go into the debate about whether that's a thing. I may have a situation, and if I'm managing it and doing well in the world, then you could say my wellbeing is is good. But my mental health in that context is extant.*
	associated with positive psychology	*mental health has emerged from psychiatry, whereas wellbeing has probably more emerged from positive psychology and the idea is that we should be looking at not just what predicts disease, but also what predicts flourishing and doing well and having a good life.*
non-clinical mental state	softer version of mental health/less clinical?	*I almost felt that wellbeing was… there was some kind of hierarchy whereby mental health was being privileged over well-being. That wellbeing somehow was perceived as softer. I may have misinterpreted that but.*
	generic feeling of a good state	*people say, well, you can't have wellbeing without having good mental health and vice versa. And there are many cases where people say, well, that's not true. You can you can have anxiety, but still live, feel like your wellbeing is good for the most part. So I think sometimes it's helpful to um, compare with the term physical health as well as physical wellbeing.*
	associated with participatory arts, not deficits	*I just wanna say like in the in the like participatory arts research, particularly with young people, they really. There really the focus is on wellbeing and I think it's like from a sort of that positive kind of philosophy and avoiding that that talk talk about deficits*
**Young people**	**Sample of codes**	***Quotes***

**Descriptive themes**		
positive attitude	associated with attitude	*having a positive mental attitude*
	associated with positive self-evaluation	*Feeling good and your judgement of life*
	forward with optimism	*I chose Forget You by CeeLo Green because it implies wellbeing because he's putting away the pains of his past and he's moving forward in a positive and happy way and overall the song is just really nice and happy.*
affective state	mood and emotions	*This relates to your mood and emotions.*
	moving on from pain	*I chose Forget You by CeeLo Green because it implies wellbeing because he's putting away the pains of his past and he's moving forward in a positive and happy way and overall the song is just really nice and happy.*
physical and mental comfort	physical and mental comfort'	*The combination of physical and mental comfort, both of which needing to be positive to have a positive wellbeing*
